# Black phosphorus, an advanced versatile nanoparticles of antitumor, antibacterial and bone regeneration for OS therapy

**DOI:** 10.3389/fphar.2024.1396975

**Published:** 2024-04-25

**Authors:** Lihui Sun, Yu Han, Yao Zhao, Jing Cui, Zhiguo Bi, Shiyu Liao, Zheru Ma, Fengxiang Lou, Chunsheng Xiao, Wei Feng, Jianguo Liu, Bo Cai, Dongsong Li

**Affiliations:** ^1^ Division of Bone and Joint Surgery, Center of Orthopedics, First Hospital of Jilin University Changchun, Changchun, China; ^2^ Jilin Provincial Key Laboratory of Oral Biomedical Engineering, School and Hospital of Stomatology, Jilin University, Changchun, China; ^3^ Department of Hepatobiliary and Pancreatic Surgery, General Surgery Center, The First Hospital of Jilin University, Changchun, China; ^4^ Key Laboratory of Polymer Eco-materials, Changchun Institute of Applied Chemistry, Chinese Academy of Sciences, Changchun, China; ^5^ Department of Diagnostic Ultrasound of People's Liberation Army 964 Hospital, Changchun, China

**Keywords:** OS therapy, black phosphorus, nanomaterials, nanomedicine, versatile therapeutic strategies, bone tissue engineering

## Abstract

Osteosarcoma (OS) is the most common primary malignant bone tumor. In the clinic, usual strategies for OS treatment include surgery, chemotherapy, and radiation. However, all of these therapies have complications that cannot be ignored. Therefore, the search for better OS treatments is urgent. Black phosphorus (BP), a rising star of 2D inorganic nanoparticles, has shown excellent results in OS therapy due to its outstanding photothermal, photodynamic, biodegradable and biocompatible properties. This review aims to present current advances in the use of BP nanoparticles in OS therapy, including the synthesis of BP nanoparticles, properties of BP nanoparticles, types of BP nanoparticles, and modification strategies for BP nanoparticles. In addition, we have discussed comprehensively the application of BP in OS therapy, including single, dual, and multimodal synergistic OS therapies, as well as studies about bone regeneration and antibacterial properties. Finally, we have summarized the conclusions, limitations and perspectives of BP nanoparticles for OS therapy.

## 1 Introduction

Osteosarcoma (OS) is the most common malignant bone tumor, accounting for approximately 56% of bone tumors (Rojas et al., 2021). The incidence of OS is 2–3 cases/million/year in the general population, but is higher in adolescents, with the highest annual incidence of 8–11 cases/million/year in 15–19 year olds (Bessen et al., 2019). The current standard treatment for OS includes extensive surgical resection, neoadjuvant chemotherapy and adjuvant radiotherapy (Thanindratarn et al., 2019). Since the introduction of chemotherapy for OS in the late 1970s, OS patients have received neoadjuvant therapy followed by a combination of postoperative adjuvant chemotherapy, i.e., elevated-dose methotrexate (12 g/m^2^), etoposide and ifosfamide for children and young adults (<25 years) in the French OS2006/Sarcome-09 study (Gaspar et al., 2018) or other regimens combining doxorubicin (DOX), cisplatin and ifosfamide with or without high-dose methotrexate (Meyers et al., 2005; Bishop et al., 2016) In order to remove the tumor totally, a large amount of tissue needs to be removed during limb-sparing surgery, which is a huge disruption to the body’s structure. At the same time, neoadjuvant radiotherapy and chemotherapy damage normal human tissue while destroying OS tissue, and these methods are not reconstructive. Under these protocols, the 5-year survival rate for children and young adults with limited OS lesions has reached 78% (Heng et al., 2020), but the survival rate for patients with metastases or recurrent OS was still 20% (Gaspar et al., 2018). Furthermore, over the previous 40 years, neither the survival rates for patients without metastases nor the survival rates for those who do have metastases have appreciably improved (Allison et al., 2012). Consequently, OS therapy continues to be a worldwide problem. Thus, the quest for the perfect biomaterial that can simultaneously eradicate tumor cells and encourage bone rebuilding is critical (Ma et al., 2020).

**FIGURE 1:**
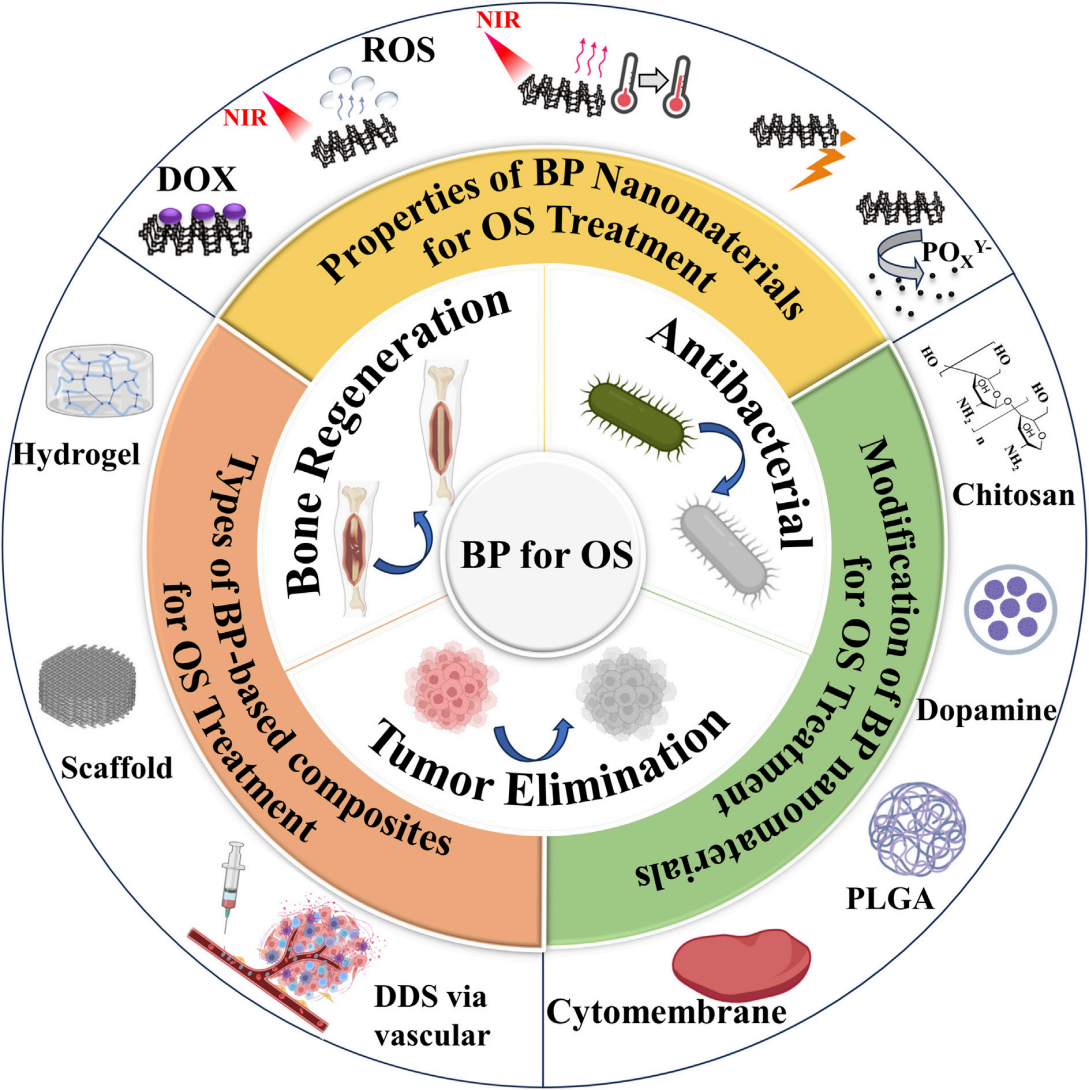
Illustration of BP for OS therapy.

Fortunately, a variety of materials have emerged for OS therapy, with previous research focusing on polymer (He et al., 2022b). These polymers require multiple modifications to be effective in OS therapy, but do not have a significant advantage in promoting bone generation and have weak mechanical properties. 2D nanomaterials are a class of emerging materials that have garnered significant attention from the scientific community. Their unique properties, such as high surface-to-volume ratio, ease of functionalization, and photothermal conversion capabilities, making them highly versatile and suitable for a large number of applications in fields such as optoelectronics and biomedical research. Some examples of these 2D nanomaterials include transition metal dichalcogenides (TMDs), transition metal oxides (TMOs), transition metal carbides, nitrides and carbonitrides (MXenes), BP, layered double hydroxides (LDHs), 2D metal-organic frameworks (MOFs), nanoclay, hexagonal boron nitride (hBN), among others (Murali et al., 2021). When combined with nanomedicine techniques, these new 2D materials hold great potential for cancer therapy, drug delivery, antimicrobial/antimicrobial platforms and tissue engineering (Zeng et al., 2022). The distinct optical or X-ray attenuation properties exhibited by 2D nanomaterials can be utilized for phototherapy (PTT) or radiotherapy in cancer treatment. Furthermore, through integration with other functional nanoparticles or leveraging their intrinsic physical properties, 2D nanomaterials can also serve as effective nanoprobes for multi-modal imaging of tumors. Notably promising examples of 2D nanomaterial-based cancer therapeutics include BiOCl/Bi_2_O_3_NSs (Chen et al., 2022a), FeOCl/FeOOH NSs (Kang et al., 2022), As/AsxOy NSs (Kong et al., 2021). In recent years, BP nanoparticles has been used in a wide range of biomedical applications, such as bone tissue engineering (Qiu et al., 2024), wound healing (Bai et al., 2024), cancer therapy (Wu et al., 2024a), epilepsy treatment (Yang et al., 2024), depression treatment (Tan et al., 2024), spinal cord rehabilitation (Liu et al., 2024), antibiotic treatment (Wu et al., 2024b). BP nanoparticles possess excellent biofouling and molecular loading capabilities for use in anticancer therapy. Additionally, their high photothermal conversion efficiency and extinction coefficient make them suitable as photothermal agents (PTAs) in PTT, where localized hyperthermia is generated to eliminate tumors through thermal means. Furthermore, BP nanoparticles can generate singlet oxygen, enabling their application as photosensitizers in photodynamic therapy (PDT) (Qing et al., 2020b). Under physiological conditions, these nanoparticles can be oxidatively degraded into non-toxic phosphonates and phosphates, promoting bone regeneration and eliminating intracellular ROS. In addition, BP nanoparticles have demonstrated the ability to modulate the bone immune microenvironment and promote osteogenesis. Recent studies have shown that BP-based PTT can activate immune responses and alleviate immunosuppression within the tumor microenvironment by detecting T lymphocytes and various immune cytokines. This suggests that BP nanoparticles not only serve as effective PTAs for ablating large solid tumors but also function as immunomodulators for eliminating smaller tumors (Liu et al., 2021a). Therefore, BP nanoparticles offer greater potential for cancer therapy compared to other 2D nanomaterials. In addition, with its features of PTT, PDT, photoacoustic therapy (PAT), drug delivery system (DDS) and biological, BP nanoparticles would become a promising material for OS therapy (Figure 1) (Raucci et al., 2019a; Zhang et al., 2019; Wang et al., 2020b; Hu et al., 2022a; Ma et al., 2022a; Dong et al., 2022; Hou et al., 2022; Li et al., 2023b; Li et al., 2023d).

## 2 Types of BP nanoparticles

**FIGURE 2:**
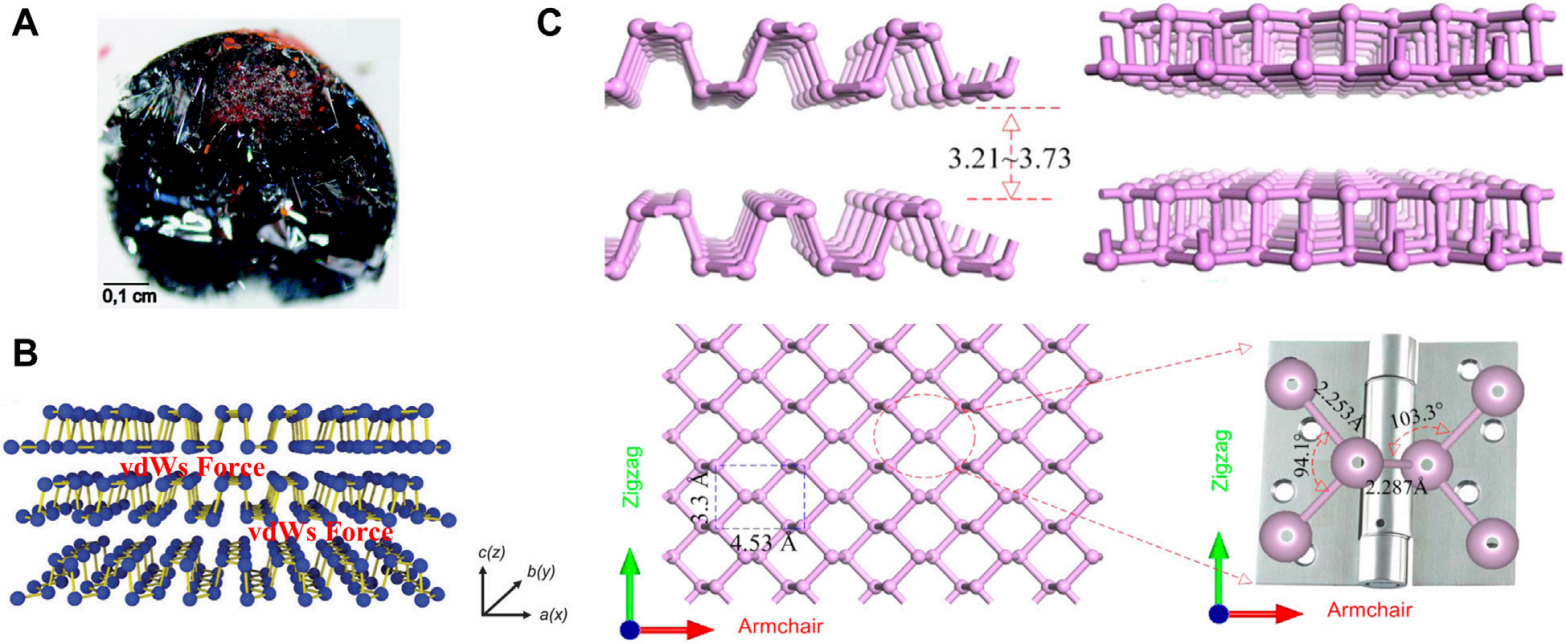
**(A)** A bulk BP crystal (scale bar = 0.1 cm). **(B)** Layered structure of BP with Van der Waal forces. (Copyright 2015 (Liu et al., 2015)). **(C)** Side views, top view of layered BP and the enlarged view of the local atomic structure of the P-P bonded configuration. (Copyright (Kou et al., 2015)).

Phosphorus has an atomic number of 15 and is in the third period of the periodic table, group VA. Phosphorus is widely distributed in nature, containing 0.12% in the Earth’s crust (Liu et al., 2015), and 0.001–0.1 mg/L in seawater (Wheat et al., 1996) and is the sixth most abundant of all elements in the human body (Calvo and Lamberg-Allardt, 2015). Phosphorus accounts for 1% of the total body mass of humans, with adults containing approximately 660 g of phosphorus, mostly in the form of hydroxyapatite in bones and teeth. In addition, phosphorus plays an essential physiological role as a major component of genetic materials, such as nucleic acids, in sustaining life, transmitting neural stimuli, and catalyzing reactions (Lee et al., 2016). Phosphorus mainly exists three forms of allotropes, including white phosphorus (WP), red phosphorus (RP), and BP (Goodman et al., 1983; Aykol et al., 2017). Among them, BP is the most stable form (Khandelwal et al., 2017). The bulk crystal of BP consists of individual layers. The crystal structure has been illustrated in Figure 2A. The layers of the BP are connected by Van der Waal forces (Figure 2B) (Liu et al., 2015) The interlayer gap varies between 3.21 Å and 3.73 Å (Figure 2C). (Kou et al., 2015) Due to the weak Van der Waal forces, the crystal thickness can be scaled down to the atomic layer scale to obtain phosphorene (or BP nanosheets (BPNSs) and BP nanoribbons (BPNRs) (Watts et al., 2019)) in 2D plane (Liu et al., 2015; Liu et al., 2021b), or even Zero-dimensional (0D) BP quantum dots (BPQDs) (Zhang et al., 2015).

Phosphorene is single or multi-layered counterpart of BP, which is 2D layered material like graphene (Liu et al., 2014a). Recently, Zhang et al. reviewed the synthesis, properties and applications of phosphenes and provided an outlook on further developments and challenges faced by phosphenes (Zhang et al., 2024). Phosphorene has a pleated structure along the armchair, but a double structure along the zigzag direction. The lattice constants in the two vertical directions are 3.30 Å and 4.53 Å (Figure 2C). (Kou et al., 2015) This structure is similar to a hinge network. The anisotropy of this structure can be distinctly seen in its local bond configuration. Zigzag direction bond angle is 94.1° and the adjacent P-P bond length is 2.253 Å. It is noted that these values are smaller than the corresponding dihedral angle (103.3°) and connected bond length (2.287 Å) along the armchair direction (Figure 2C). (Kou et al., 2015) Since phosphorene was successfully prepared in 2014, there has been an explosion of research on it, revealing that it has considerable potentiality for application in a vast number of fields (Liu et al., 2014a). Apart from graphene, phosphorene is the only stable 2D monolithic material that can be mechanically exfoliated. Unlike graphene, it has inherent, direct, and appreciable band gap (Liu et al., 2014a). Phosphorene includes BPNSs (Thurakkal and Zhang, 2019; Liu et al., 2021b) and BPNRs (Watts et al., 2019). Of all the phosphorene used in OS therapy, BPNSs were the most widely used because of their superior photothermal conversion efficiency and high surface area-to-volume ratio. In early vitro cellular studies have demonstrated that BPNSs can alleviate oxidation-induced apoptosis by clearing reactive oxygen species (ROS) (Liu et al., 2021b). Besides, when combined with near-infrared radiation (NIR), BPNSs trigger a photothermal response that leads to high local temperatures and can effectively ablate tumor cells (Ma et al., 2022a; Li et al., 2023b).

Except BPNSs, BPQDs are also the widely explored nanoplatform for OS therapy. *In vivo* experiments have proven that BPQDs are non-toxic (Mu et al., 2017). Similar to BPNSs, BPQDs also have good photothermal conversion efficiency and have been used in PTT of tumor (Zhang et al., 2019; Hu et al., 2022a). Unlike the BPNSs, BPQDs are much smaller in diameter, averaging around 10 nm. The smaller size facilitates the distribution of BPQDs in the body and allows them to be protected from phagocytosis by macrophages, extending the half-life of BPQDs (Pandey et al., 2021).

## 3 General synthesis of BP nanoparticles

In general, BP nanoparticles are mainly synthesized by top-down methods. The top-down method is the exfoliation of bulk BP into BP nanoparticles by physical or chemical routes. Based on the weak van der Waals forces in BP nanoparticles, it is easy to convert bulk BP into BP nanoparticles. Therefore, we provided a detailed review of synthesis methods for BP nanoparticles in this section.

### 3.1 Mechanical exfoliation method

**FIGURE 3:**
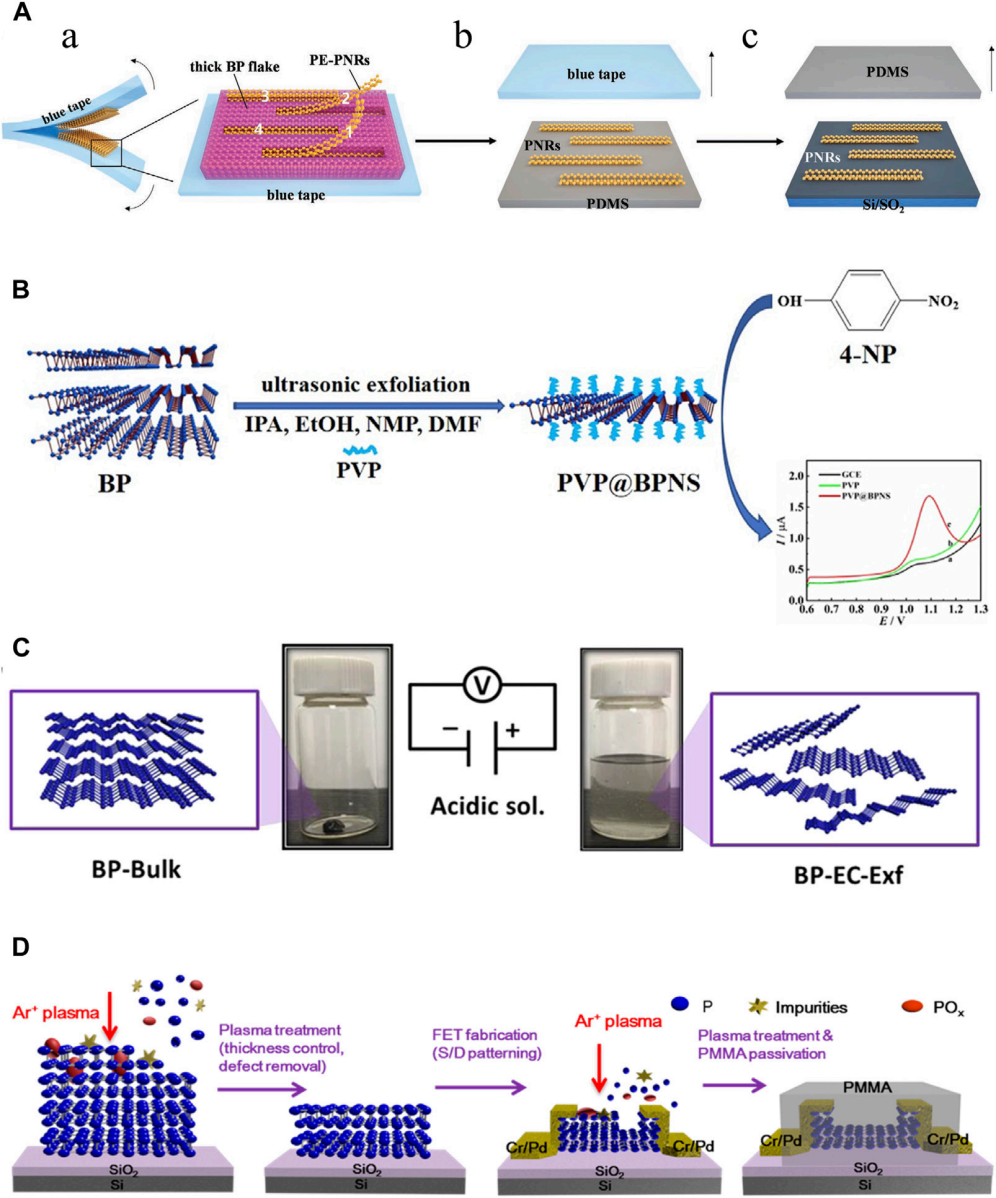
Synthesis of BP nanoparticles. **(A)** Mechanical exfoliation. **(A)** Preparation of the partially-exfoliated PNRs (PE-PNRs) connected to a thick BP fake via the blue tape exfoliation (left) and the zoomed-in view of the exfoliated product (right). **(B)** The PE-PNRs were further peeled off to obtain separated individual PNRs on the polydimethylsiloxane (PDMS) substrate by the PDMS exfoliation. **(C)** The resulting PNRs in **(B)** were transferred from the PDMS substrate onto the SiO_2_/Si substrate by the conventional dry transfer way. (Copyright (Hu et al., 2023)). **(B)** Liquid exfoliation. BPNSs are simply prepared through ultrasonic exfoliation of bulk BP with the assistance of PVP in different solvents, including IPA, EtOH, NMP and DMF. (Copyright (Shen et al., 2021)). **(C)** Electrochemical method. The bulk BP crystal is exfoliated in a phytic acid solution by applying a positive voltage. Bulk BP crystals (left) and exfoliated BP-EC-Exf (right) in the solvent. (Copyright (Qiu et al., 2019)). **(D)** Plasma etching method. Schematic diagram of the effects of the plasma treatment of a BP flake: thickness control, surface defect removal, and device fabrication process. (Copyright (Jia et al., 2015)).

Mechanical exfoliation was the earliest method to produce BP nanoparticles. In 2014, Xia et al. mechanically exfoliated BPNSs from bulk BP crystals using a 300 nm SiO_2_ grid (Xia et al., 2014). Meanwhile, Liu et al. also demonstrated that atomic-scale monolayer/multilayer BP nanoparticles can be obtained by mechanical exfoliation (Liu et al., 2014b). However, the mechanical exfoliation method at this time had some drawbacks such as low efficiency, product instability, lack of scalability, adhesive residue and difficulty in controlling the number of layers of BP nanoparticles. To upgrade the traditional mechanical exfoliation method. Metal or plasma-assisted mechanical exfoliation methods emerged and were developed. Gaun et al. prepared a large number of phosphene layers with sizes exceeding 50 μm by combining Au/Ag on a SiO_2_/Si grid to cut bulk BP (Guan and Xing, 2018). Lu et al. first combined mechanical exfoliation with Ar + plasma to obtain stabilized BPNSs with one to five layers (Lu et al., 2014). The later study by Kuriakose et al. showed that O_2_ plasma is more suitable than Ar + plasma for mechanical exfoliation of BP nanoparticles (Kuriakose et al., 2018). Recently, Hu et al. invented a modified mechanical stripping method of tape stripping to prepare narrow and edge-flattening black phosphorus nanoribbons (PNRs). (Figure 3A). (Hu et al., 2023) With these improvements, mechanical exfoliation methods have been greatly enhanced for use in the industrial and pharmaceutical industries.

### 3.2 Liquid exfoliation method

The liquid exfoliation method is currently the most commonly used technique for large-scale production of BP nanoparticles (He et al., 2024). Immersion of bulk BP crystals in an organic solvent creates ionic exchange between BP layers, which causes bulk BP to exfoliate and form monolayer BPNSs. The forces between the crystal structures of the bulk BP become weak in the solvent, and then the BPNSs are obtained by different methods (ion insertion, ultrasound-assisted, ion-exchange, and shear-exfoliation). The key to the liquid exfoliation method is the choice of solvent. Liquid solvents include N-methyl-2-pyrrolidone (NMP), N-cyclohexyl-2 (or3)-pyrrolidone (NCP), dimethyl sulfoxide and dimethylformamide (DMF), NMP/NaOH mixture and organic/ionic mixture. Unlike mechanical exfoliation, liquid exfoliation can protect the BP nanoparticles from oxidation. Another consideration is to avoid degradation of BP nanoparticles in liquid solvents. In general, solvents with strong surface tension and high dielectric constants can reduce BP degradation in liquids. Shen et al. prepared BPNSs by ultrasonic stripping of bulk BP in different solvents such as iso-Propyl alcohol (IPA), ethanol (EtOH), and DMF using polyvinylpyrrolidone (PVP) as an auxiliary agent, there was found that the stripping efficiencies of IPA and EtOH were much higher than DMF and NMP, and the stripping efficiencies of IPA were better than EtOH. More importantly, PVP reduces the oxidation of BPNSs by forming a protective film, which effectively improves the productivity and environmental protection (Figure 3B). (Shen et al., 2021) In a review, it was detailed that biomolecules such as peptides, proteins, nucleic acids, *etc.*, have been used as exfoliators for the synthesis of high-quality graphene, which opens up possibilities for the production of high-quality BP nanoparticles (Paredes and Villar-Rodil, 2016). Recently, Yang et al. proposed a new synthesis strategy of BPNSs, which used Tween 20 assisted deoxygenation water to efficiently prepared fewer or even single layers of BPNSs (Yang et al., 2020). However, the liquid exfoliation stripping method also has the disadvantages of organic media contamination and time-consuming, and ultrasound-induced damage to the crystal structure can hinder its use in electronics.

### 3.3 Electrochemical method

Indeed, the electrochemical method belongs to one of the classes of liquid exfoliation methods. The electrochemical method is simple, cost-effective and environmentally friendly methods that has been widely used to prepare 2D nanoparticles such as graphene with satisfactory results. The electrochemical method can be used to synthesize BPNSs on a large scale, which has the advantages of high product yield, high crystalline quality of samples, rapid synthesis process without any additional catalysts, which is commonly used in the fabrication of nano-electronic components. Traditionally, bulk BP is the anode, platinum wire is the cathode, and sulfuric acid solution is used as the electrolyte in the electrochemical methods. With the applied voltage, the BP crystal gradually peels away to form BPNSs. However, when BP crystals are used as the anode, the product is prone to oxidation. Later, Xiao et al. invented an electrochemical cathodic stripping method, in which BP was used as the cathode, a platinum plate as the anode, and tetrabutylammonium hexafluorophosphate as the electrolyte, obtaining BPNSs with a thickness of 2–7 nm, and oxidation could be avoided completely in this process (Xiao et al., 2018). Due to the bare surface nature of this electrochemically stripped BP, it is difficult to disperse and is not compatible with common polymer substrates. To overcome this difficulty, the surface functionalization of graphene has been inspired. Qiu et al. used BP crystal as cathode and phytic acid as electrolyte to generate BPNSs functionalized with cobalt phytate (BP-EC-Exf), and introduced BP-EC-Exf into polyurethane acrylate (PUA) matrix by ultraviolet (UV) curing strategy to prepare superior-performance PUA/BP-EC-Exf (PUA/BP-EC) nanocomposites (Figure 3C) (Qiu et al., 2019).

### 3.4 Plasma etching method

The plasma etching method involves placing BP crystals on a silica substrate and exposing them to an oxygen plasma through which the upper layer is oxidized to a PxOy layer, resulting in the separation of the BP crystals and the formation of an arbitrary number of layers of BPNSs. The PxOy layer protects the BPNSs from oxidation during this process. Jia et al. fixed BP crystals on a silicon plate, peeled off the bulk BP crystals with blue Nitto tape, and then treated them with Ar + plasma to obtain BP films with a controlled number of layers, utilized the BP films to fabricate BP field effectiveness transistors (FET), and finally passivated the FET with polymethylmethacrylate (PMMA) (Figure 3D). (Jia et al., 2015) However, this technique has rarely been investigated further in recent years.

## 4 Properties of BP nanoparticles for OS therapy

In the monolayer BP structure, each phosphorus atom is linked to three neighboring phosphorus atoms by covalent bonds, forming a folded structure. This folded structure endows the BP nanoparticles with high surface area-to-volume ratio, excellent mechanical, optical, thermal and electrical properties (Zhang et al., 2021). Based on the excellent properties, BP nanoparticles have been used in many fields. In this section, we only summarized the properties of BP nanoparticles in the field of OS therapy (Table 1). The wide and adjustable bandgap of BP nanoparticles creates high light absorption efficiency. Moreover, the high photothermal conversion efficiency allows for a good performance of photothermal conversion. Hyperthermia leads to the thermal elimination of tumor cells; thus, BP nanoparticles can be applied as a photothermal agent for PTT. In addition, BP nanoparticles can also be used as a photosensitizer for PDT of OS therapy. Under the NIR, BP nanoparticles can transfer energy to the surrounding oxygen to release ROS, including superoxide anion radicals, hydrogen peroxide, highly reactive hydroxyl radicals, and single-linear oxygen species, which kill tumor cells. ROS can also act as an antibacterial agent to prevent infections in OS therapy (Li et al., 2023d). Besides, the localized warm environment promotes bone regeneration by activating several osteogenic proteins including alkaline phosphatase (ALP) and heat shock protein (HSP).

**TABLE 1:** Important properties of BP nanoparticles for OS Therapy.

Properties	Exhibited by BP	Related applications
Bandgap	0.3–2.0	The wider bandgap range gives BP good optical absorption and photothermal conversion, which makes BP a popular choice for tumor treatment. (Liu et al., 2021c; Zhong et al., 2021)
Photothermal conversion efficiency	high	
Surface-area-to-volume ratio	large	BP nanoparticles have a large surface-area-to-volume ratio and contain targets for anchoring chemotherapeutic drugs, which can be efficiently used as tumor drug carriers as well as for photothermal-chemotherapy therapy of tumors. (Ma et al., 2022a)
Biocompatibility	Good	BP nanoparticles has good biocompatibility and relatively low cytotoxicity. (Dong et al., 2022)
Biodegradability	Good	BP nanoparticles can be degraded *in vivo* to non-toxic phosphate, which are good for bone regeneration. (Jing et al., 2022)
Electrical conductivity	Good	Black phosphorus nanoparticles have excellent electrical conductivity and are bipolar, which is beneficial for nerve regeneration. (Xu et al., 2022b; Li et al., 2023a)

BP nanoparticles have a folded structure that gives them a large surface area-to-volume ratio, allowing them to be efficiently loaded with tumor agents. Chen et al. adsorbed DOX onto BPNSs via electrostatic interactions and showed that the drug loading rate of BPNSs reached 950%, which is higher than any previously reported 2D nanoparticles (Chen et al., 2017). Alternatively, DDS can be combined with PTT to form a photothermal-thermal therapy (Li et al., 2023b; Xu et al., 2023). BP nanoparticles can also form microspheres that encapsulate the drug and reach deeper tumor locations, forming a multimodal therapy of tumors through synergistic PDT and immunotherapy (Li et al., 2019). BP nanoparticles are degradable and their degradation product is phosphate, which is non-toxic to cells. Phosphate is also one of the main components of bone and can promote bone regeneration by trapping calcium to form calcium phosphate (CaP) deposits (Qing et al., 2020a). Moreover, a sudden increase in the concentration of intracellular phosphate can also lead to oxidative stress in tumor cells, which ultimately leads to apoptosis (Zhou et al., 2019). BP nanoparticles also have excellent electrical conductivity and facilitate nerve regeneration during bone regeneration (Qian et al., 2019).

## 5 Modification of BP nanoparticles for OS therapy

Applications for BP nanoparticles in biomedicine are numerous. However, the BP nanoparticle’s instability is currently the issue. It is imperative that BP nanoparticles be modified in order to increase their stability. Recently, An et al. reviewed the progress of polymer-decorated BP nanoparticles for biomedical therapeutic applications (An et al., 2020). Besides, the strategies and recent advances in surface modification of BP nanoparticles for biomedical applications can be accessed through the review of Zeng et al. (Zeng and Chen, 2020) Here we summarize some modification strategies of BP nanoparticles for OS therapy.

### 5.1 Electrostatic adsorption of cationic polymers

BP nanoparticles have negative potentials and can easily adsorb cationic polymers via electrostatic interactions. Currently, cationic polymers used to enhance the stability of BP nanoparticles in OS therapy include chitosan (CS) and dopamine (DA).

#### 5.1.1 Chitosan

CS, as a cationic polysaccharide, has good biocompatibility, biodegradability and antibacterial properties (Zhao et al., 2022b). CS promotes osteogenic differentiation by upregulating the expression levels of Osterix and bone sialoprotein (BSP). Electrostatic interactions between positively charged groups on CS and negatively charged groups on the bacterial cell wall can kill bacteria by cutting off their nutrient and oxygen supply. Hydrophobic interactions between the CS and bacteria also have a bactericidal effectiveness. Moreover, the abundant functional groups (hydroxyl and amine) in CS can act as donors and acceptors to promote the formation of intramolecular and intermolecular hydrogen bonds between hydroxyl and amine in DOX, which facilitates the delivery of DOX (Hosseini et al., 2022). Positively charged CS molecules can be adsorbed onto negatively charged BPNSs via electrostatic interactions, which have been shown to enhance the stability of BP nanoparticles in physiological settings. Therefore, CS-modified BP nanoparticles are highly promising for constructing DDS for OS therapy in combination with osteogenesis and antibacterial. There are *in vitro* and *in vivo* studies that show: the result of antibacterial and antitumor can be accomplished in one step at a temperature of 49 ± 0.5°C due to the synergistic effectiveness of hydroxypropyl-trimethyl ammonium chloride CS and thermotherapy. At a temperature of 42 ± 0.5°C, NIR can promote osteogenesis (Zhao et al., 2023b). Recently, He et al. designed a multilayered assembled composite coating consisting of negatively charged BPNSs and positively charged CS for 3D printing of polyaryletherether ketone (PEEK) bone scaffolds (He et al., 2022a). The BPNSs in the composite coating inhibited infection via PTT, while the pH-sensitive CS acted as a “smart” DDS. The CS polymer also effectively protects the BP nanoparticles from rapid degradation, allowing for the sustained release of PO_4_^3-^, which promotes bone regeneration. In this experiment, pristine BPNSs were completely degraded within 1 h, and BP crystals were fully degraded after 440 h. BPNSs encapsulated in CS further inhibited their degradation rate, and they were completely degraded only after 1,351 h (56 d). The results showed that the release of both phosphate and DOX was enhanced at pH 5.5, indicating good pH control of drug release. This may be due to the protonation of CS amino groups in an acidic environment, and polymer swelling can expand the envelope porosity and promote drug release through enhanced diffusion.

#### 5.1.2 Dopamine

The high adhesion capacity of mussels is due to *Mytilus edulis* foot protein (MEFP), which includes DA and its derivatives polydopamine (PDA), is a promising coating in the biological field. PDA coatings can be firmly adhered to the surface of numerous materials and further amine/thiol coupling to secondary biopolymers via Michael additive or Schiff base reactions. It has been demonstrated that PDA has strong NIR absorption and high photothermal conversion efficiency. Therefore, the PDA wrapping BPNSs not only improves the stability of BPNSs by isolating them from the external environment, but also enhances the photothermal conversion efficiency of BPNSs. Ma et al. showed that by encapsulating BP nanoparticles with PDA, the PDA layer enhanced the photothermal properties of BP nanoparticles and that the hydrophilic and biocompatible nature of the PDA layer enhanced cell adhesion and conferred the NIR/PH dual-responsive DOX-release properties of this therapeutic system (Ma et al., 2022b). Recently, researchers found that the irradiation with an 808 nm laser (1W cm^−2^, 10 min) raised the temperature of the BPNSs solution by 6.3°C when the BPNSs were bound with the PDA films, suggesting the PDA coating effectively enhanced the photothermal activity of BPNSs (Li et al., 2023d). Interestingly, BPNSs protected by PDA (BPNSs@PDA) showed the highest drug release than bare BPNSs, presumably due to the photothermal additive effectiveness of PDA, the higher the temperature, the weaker the electrostatic attraction and the easier the DOX release. *In vivo*, this integrated system achieves a good photothermal conversion efficiency via BP@PDA, killing bacteria in the initial phase by PTT of BPNSs, and finally killing tumor cells and promoting osteogenesis in the later phase by PTT of BPNSs in synergy with DOX (Li et al., 2023d).

### 5.2 Polymer vesicle encapsulation

Polymer vesicles are micro to nanoscale polymer capsules with bilayer membranes. Poly (lactic-co-glycolic acid) (PLGA) is a linear copolymer of lactic and glycolic acid monomers, one of the most widely used biodegradable polymers. PLGA has excellent biocompatibility, well processability, gradual degradation property and can be loaded with a variety of bioactive factors; therefore, it is often used in drug delivery, bone regeneration, and tumor therapy (Zhao et al., 2021; Feltrin et al., 2022; Lim et al., 2022). BP nanoparticles/PLGA composites were formed when PLGA encapsulated BP nanoparticles, and the BP nanoparticles/PLGA composites had strong hydrophobicity, good biocompatibility and no appreciable toxicity (Shao et al., 2016). Owing to the well hydrophobicity of PLGA, rapid degradation of the BPQDs is prevented, so the BP nanoparticles/PLGA composites possesses good PTT efficiency and tumor targeting ability (Shao et al., 2016).

Wang et al. (Wang et al., 2020a) prepared PLGA-encapsulated BPNSs nanospheres (BPNSs@PLGA) by oil-in-water method for synthetic composite scaffolds for OS therapy and found that the scaffolds could maintain the photothermal conversion capability for more than 3 weeks, which was due to the reduced decomposition rate of BPNSs, as most of the BPNSs were embedded in the polymer matrix and degraded only with the hydrolysis of PLGA, showing a much longer window of time for photothermal therapy operation as compared to other studies in which the BPNSs were deposited only on the surface of the scaffolds (Yang et al., 2018). Recently, Hu et al. (Hu et al., 2022a) encapsulation of BPQDs in PLGA by oil-in-water emulsion solvent evaporation method to form BPQDs/PLGA nanospheres (NSs) and combined with wood and filipin proteins to synthesize hydrogels for the therapy of bone tumors. TEM and FE-SEM results show that BPQDs/PLGA NSs have regular shapes and large radii. Statistical analysis of TEM and AFM data on a sample of 100 BPQDs yielded an average size of 2.39 nm and thickness of 2.21 nm, respectively. The average hydrodynamic size of the BPQDs/PLGA NSs was centered at 139.1 nm, which was larger than the size of the BPQDs, suggesting the PLGAs successfully encapsulated the BPQDs. It was demonstrated by Raman spectroscopy that BPQDs/PLGA NSs were more stable than BPQD in horseradish peroxidase (HRP)/H_2_O_2_ solution. Comparing the Raman spectra at 0 and 8 days, no significant difference was recorded in the BPQDs/PLGA NSs, but there was a significant blue shift in the Raman spectra of the BPQDs. In addition, the absorption spectra of both BPQDs and BPQDs/PLGA NSs exhibited a typical broad absorption range across the UV and NIR regions. However, when dispersed in water with HRP/H_2_O_2_, the absorption intensity of the BPQDs decreased with dispersion time. In summary, the hydrophobicity of PLGA, which protects BPQDs from rapid oxidative degradation, can allow BPQDs to degrade slowly. At the same time, BPQDs/PLGA NSs exhibit stronger absorption strength and drug carrying capacity than BPQDs due to their larger size.

### 5.3 Cytomembrane encapsulation

Recently, the biomimetic modification strategies of biofilm-encapsulated nanoparticles have gained more and more acceptance. In 2011, Hu et al. first reported the use of erythrocyte membranes to encapsulate PLGA nanoparticles for long-period cargo delivery (Hu et al., 2011). It provides a new idea for the modification of nanoparticles. In later studies, biofilms were widely used in tumor therapy (Zhang et al., 2022b; Choi et al., 2023). It has been recognized that biofilms are non-cytotoxic and highly bioavailable, and that cell membranes carrying cellular immunomodulatory self-labeling proteins can provide the encapsulated nanoparticles with anti-phagocytic activity, prolonged lifespan *in vivo*, and targeted delivery of the nanoparticles to the lesion, thus enhancing the effectiveness of tumor therapy. Currently, biofilms prepared from various cell membranes (erythrocytes, leukocytes, stem cells, and tumor cells) have been used to encapsulate nanoparticles individually or in a fusion fashion for complex tasks in various physiological environments. Erythrocyte membranes allow the encapsulated nanoparticles to evade immune surveillance, leukocyte membranes are resistant to the action of conditioning agents, stem cell membranes have a naturally high tumor affinity for targeted delivery, and tumor cell membranes have homologous recognition properties. Unfortunately, none of the aforementioned cell membrane encapsulated BP nanoparticles have been reported for OS therapy. The platelet membrane surface contains CD_47_, which interacts with signal-regulating proteins on immune cells to evade immune clearance and has been used in tumor therapy (Bahmani et al., 2021). Recently, Xu et al. firstly used OS cell membranes (OCM) fused with platelet cell membranes (PM) to construct platelet-OS hybrid membranes (OPMs) to encapsulate BPQDs (BPQDs@OPM), where DOX was loaded on the BPQDs (BPQDs-DOX@OPM) (Xu et al., 2023). Finally, a non-toxic, long-acting, highly effective, targeted chemotherapy in combination with PTT for OS therapy was obtained.

## 6 Types of BP-based composites for OS therapy

In OS therapy, in order to better exploit the excellent properties of BP nanoparticles, it is necessary to place BP nanoparticles on a suitable platform to form BP-based composites. Currently, BP-based composites for OS therapy include BP-based hydrogels, BP-based bone scaffolds and BP-based DDS via vascular injection.

### 6.1 BP-based hydrogel

**FIGURE 4:**
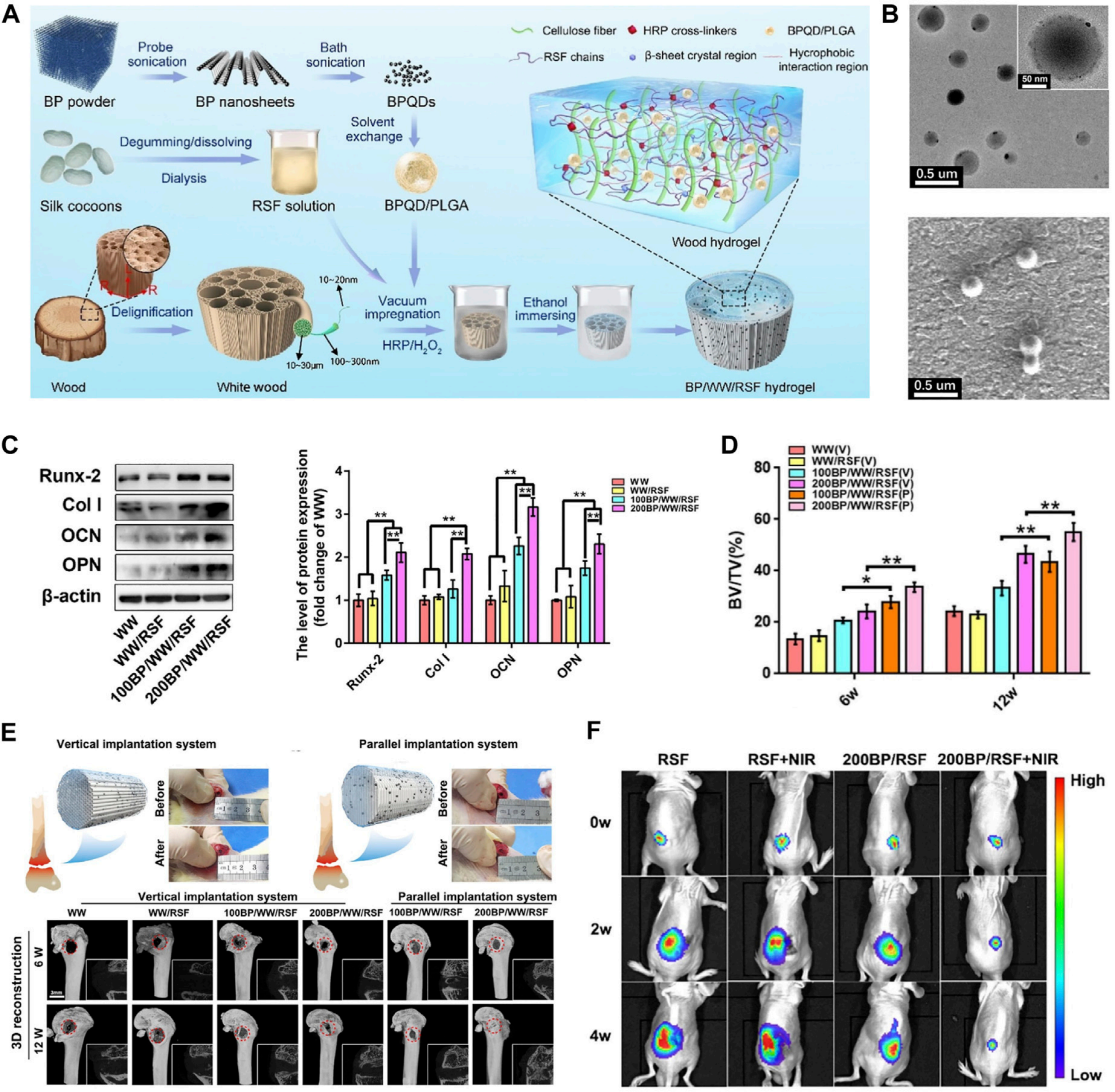
BP-based hydrogel for OS therapy. **(A)** The fabrication process of BP/WW/RSF hydrogel scaffold. **(B)** The TEM and SEM images of BPQD/PLGA NSs. **(C)** Representative Western blot data and quantitative analysis of the expression of Runx-2, Col-1, OCN, and OPN. **(D)** BV/TV results calculated based on micro-CT. **(E)** The procedure of implanting hydrogel scaffolds into the critical bone defect in rat femurs and 3D, 2D reconstruction of micro-CT at 6, 12 weeks after implantation. **(F)** Luminescence images of mice before and after treatments. (Copyright (Hu et al., 2022a)).

Hydrogels are one of the most mature materials for biomedical applications and are widely used in many biomedical fields, including tissue engineering, wound healing, drug delivery, and tumor therapy. Whereas the limited properties of conventional hydrogels hamper their potential of applications, and nanocomposite hydrogels combine the properties of nanoparticles to provide superior functionality. BP is commonly incorporated into hydrogels due to its biodegradability, biocompatibility, photothermal properties, electrical conductivity, and high surface area-to-volume ratio, which improves the mechanical strength and stimulus responsiveness of hydrogels. The photothermal conversion efficiency of BP nanoparticles soften the hydrogel, allowing for the release of hydrogel-loaded agents that can be accurately controlled by the intensity of the light, the exposure time, the BP dose, and the hydrogel composition. Hu et al. (Hu et al., 2022a) designed a high-strength WW/RSF hydrogel integrated with PLGA encapsulated BPQDs for efficient mechanical support, bone regeneration, and OS therapy (Figure 4). Better osteogenic differentiation of BMSCs, accelerated bone regeneration was observed in BP/WW/RSF systems, also had good photothermal conversion efficiency and promoted tumor ablation. BPQDs in hydrogel inhibit osteoclast differentiation and have been shown to be photothermal effective against spinal metastases. Moreover, the hydrogel is injectable and can be used for thermal ablation of OS by *in situ* injection (Li et al., 2023c). Therefore, BP-based hydrogels have frequently been reported for OS therapy in recent years. In addition to being injected *in situ* into OS lesions for thermal ablation, BP-based hydrogels can also be formed into coatings used to modify hard scaffolds for OS therapy.

### 6.2 BP-based scaffold

**FIGURE 5:**
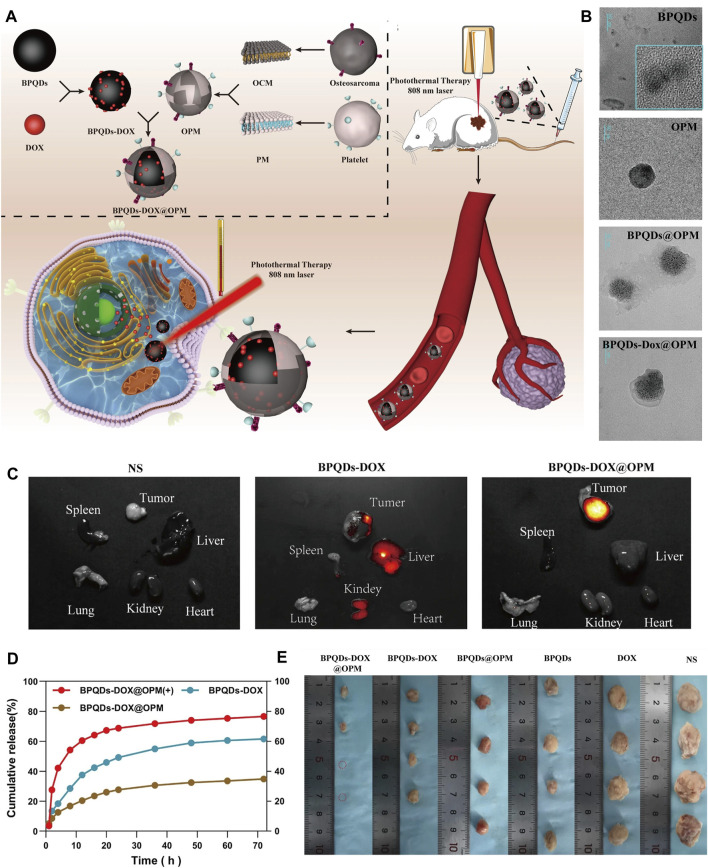
BP-based DDS for OS therapy. **(A)** Schematic diagram of the role of OPM camouflaged with BPQDs-DOX in combined treatment of OS. **(B)** Transmission electron micrographs of BPQDs, OPM, BPQDs@OPM, and BPQDs-DOX@OPM (size: 50 nm). **(C)** BPQDs-DOX@OPM *in vivo* targeting study. **(D)** DOX release characteristics of BPQDs-DOX@OPM. **(E)** Tumor tissues after 18 days of intravenous injection of NS, BPQDs, DOX, BPQDs-@OPM, BPQDs-DOX, and BPQDs-DOX@OPM. (Copyright (Xu et al., 2023)).

Bone scaffolds play a vital role in cell proliferation, differentiation and angiogenesis due to their inherent and excellent osteogenic, bone conduction and bone induction properties, and are often used in the treatment of bone defects in OS therapy. There are many bone scaffolds used in OS therapy, including bio-glass (BG) scaffolds (Yang et al., 2018), PEEK scaffolds (He et al., 2022a), PPENK scaffolds (Li et al., 2023d) and organic polymer composite scaffolds. The surface area of the scaffold provides convenience for the adhesion of BP nanoparticles, and the osteogenic effectiveness of the scaffold is coordinated with the osteogenic effectiveness of BP nanoparticles, and combined with the photothermal and photodynamic effectiveness of BP nanoparticles, which together constitute a multifunctional treatment platform for OS therapy. Recently, Ma et al. (Ma et al., 2022b) designed a multifunctional multiscale therapeutic platform (Fs-BP-DOX@PDA) (Figure 5). First, micro-nanostructures (Fs-NiTi) were fabricated using femtosecond laser direct writing technology. Then, BPNSs were added to Fs-NiTi to construct multilayer structures (Fs-BP). Finally, the DOX-loaded Fs-BP was modified using a PDA. As a result, the Fs-BP had a higher loading efficiency of 93.2% than the Fs-NiTi (35.2%) and P-NiTi (13.3%). Fs-BP-DOX@PDA effectively killed tumor cells (Saos-2 and MDA-MB-231) *in vitro*, completely eradicated OS cells of mice *in vivo* and promoted bone regeneration. In summary, BP-based bone scaffolds have promising prospects in OS therapy.

### 6.3 BP-based DDS via vascular injection

**FIGURE 6:**
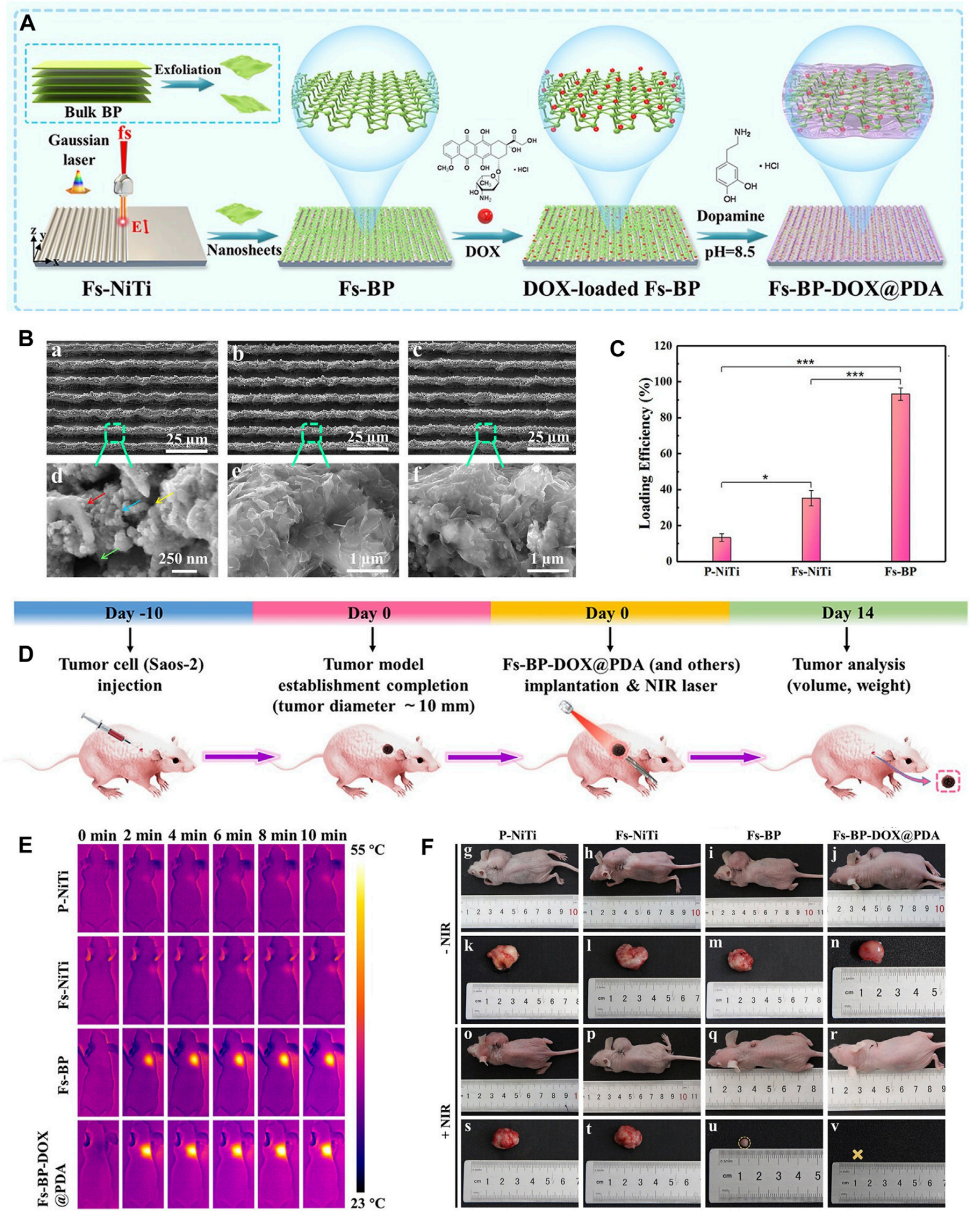
BP-based scaffold for OS therapy. **(A)** Construction procedure of the Fs-BP-DOX@PDA. **(B)** SEM images of the **(A, D)** Fs-NiTi, **(B, E)** Fs-BP, and **(C, F)** Fs-BP-DOX@PDA. **(C)** DOX loading efficiencies. **(D)** Schematic illustration of ectopic osteosarcoma model establishment and timeline of the therapy schedule. **(E)** Real-time infrared thermographs of the sample-treated tumor-bearing mice under NIR laser irradiation. **(F)** Digital images of tumor-bearing mice and the corresponding tumor sites recorded at the end of the treatment period. (Copyright (Ma et al., 2022b)).

The BP-based DDS via vascular injection has not been studied much, and offers a new idea for targeted treatment of OS. A recent study (Xu et al., 2023) has reported that BPQDs and DOX were encapsulated in OPM to build the BPQDs-DOX@OPM group to achieve a targeted comprehensive treatment strategy of PTT combined with chemotherapy for OS (Figure 6). The OPM encapsulation system also improves the stability of BPQDs by reducing their exposure to oxygen and water, thereby improving their photothermal conversion efficiency. *In vitro*, Convert the OPM encapsulation system improved stability of BPQDs and increased 72-h release of BPQDs-DOX@OPM and BPQDs-DOX to 34.88% and 61.53%, respectively. This indicated that the OPM hybrid membrane encapsulation system could slow down the release of DOX and prolong the half-life of BPQDs-DOX@OPM in the blood circulatory system. *In vivo*, after 10 min of irradiation with 808 nm near-infrared light, the temperatures of the BPQDs and BPQDs-DOX@OPM groups increased rapidly, reaching 42.7°C and 48.9°C, respectively. The tumor weight in the BPQDs@OPM group was significantly smaller than that in the BPQDs group. In conclusion, the BPQDs-DOX@OPM group was demonstrated to perform better than the bare BPQDs group. This study proposed a nontoxic, long-circulating, targeted delivery chemotherapy-PTT combined DDS for the OS therapy.

## 7 Applications of BP nanoparticles for OS therapy

### 7.1 BP nanoparticles for tumor elimination

BP is an emerging versatile nanomaterial with good biocompatibility and biodegradability, with promising applications in oncology. BP nanoparticles with high photothermal conversion efficiency can be used as photothermal agents in PTT to generate localized hyperthermia leading to thermal elimination of the tumor. In addition, BP nanoparticles are capable of generating ROS, which makes them useful as photodetectors for PDT. Moreover, BP nanoparticles have a high surface area-to-volume ratio, which improves their efficiency as DDS in tumor therapy. Excitingly, recent studies have shown that BP nanosheets have selective anti-cancer potential against osteosarcoma cells (Bigham et al., 2024). In conclusion, BP nanoparticles can form multiple therapeutic modalities for OS therapy (Table 2).

**TABLE 2:** Strategies of BP nanoparticles for OS therapy.

Modality	Platform	Tested models	Main results	Ref
PTT	3D printed BPNSs BG scaffold	*In vitro*: Saos-2 cells, hBMSCs	Situ phosphorus-driven, calcium-extracted biomineralization of the intra-scaffold BPNSs enables both photothermal ablation of OS and bone regeneration	Yang et al. (2018)
		*In vivo*: Saos-2 subcutaneous OS, skull bone defect in SD rat		
PTT	Wood/silk fibroin hydrogel scaffold with BPQDs/PLGA nanospheres	*In vitro*: A549 cells, hBMSCs	Inhibit osteoclast differentiation, exhibit photothermal effectiveness against metastatic tumor in the spine and bone regeneration	Hu et al. (2022a)
		*In vivo*: A549 cells spinal metastasis model, femoral defect in BALB/c mice		
PTT	CS/hydroxypropyl trimethyl ammonium chloride	*In vitro*: rBMSCs, MG-63, *E. coli*, *S. aureus*	Antibacterial and antitumor at a temperature of about 49°C, osteogenesis was promoted at a temperature of about 42°C	Zhao et al. (2023b)
	CS/hydroxyapatite/BP hybrid photothermal scaffold	*In vivo*: MNNG/HOS in the hip of BALB/c mice, skull bone defect in SD rat		
CPT	3D printed water/PLGA/dichloromethane emulsions containing β-tricalcium phosphate (β-TCP), BPNSs nanocomposite scaffolds	*In vitro*: rBMSCs, MG-63	Excellent tumor cell ablation, significantly improved rBMSCs osteogenic differentiation *in vitro* and enhanced regeneration of the defected rat cranial bone *in vivo*	Wang et al. (2020a)
		*In vivo*: MNNG/HOS in the hip of BALB/c mice, cranial bone defect in SD rat		
CPT	Fs-NiTi-BP- DOX @ polydopamine versatile multiscale therapeutic platform	*In vitro*: Saos-2, MDA-MB-231, MC3T3-E1, *S. aureus*, *P. aeruginosa*	Controllable drug release behavior of NIR/pH-dual sensitivity, completely eliminated OS without any recurrence *in vivo*, significantly accelerated the osteogenesis of MC3T3-E1 cells, excellent photothermal antibacterial efficiencies toward *S. aureus* (99.2%) and *P. aeruginosa* (99.6%)	Ma et al. (2022b)
		*In vivo*: Saos-2 subcutaneous OS of BALB/c mice, MDA-MB-231 cells bone metastasis model of BALB/c mice		
CPT	Injectable thermosensitive BPNSs/DOX/CS hydrogel	*In vitro*: K7M2-WT, MC3T3-E1	The combination of photothermal and chemotherapy of OS was realized, promoted osteogenic differentiation	Li et al. (2023c)
		*In vivo*: K7M2-WT in the right forelimb of BALB/c female mice		
CPT	BPQDs-DOX@ platelet-OS hybrid membrane (OPM)	*In vitro*: Saos-2	Compared to single-agent chemotherapy, the combined therapy using BPQDs-DOX@OPM offers pro-longed circulation time, targeted drug delivery, enhanced anti-tumor activity	Xu et al. (2023)
		*In vivo*: Saos-2 subcutaneous OS of BALB/c mice		
PDT	Bare few-layer BP	*In vitro*: Saos-2, HOb, hBMSCs, L929	Induction of anti-proliferative and apoptotic effectiveness in Saos-2 cells through increased ROS production	Raucci et al. (2019b)
		*In vivo*: None		
Chemotherapy combined with PTT and PDT	3D printed PEEK bone scaffold with BPNSs/CS multifunctional composite coating	*In vitro*: MG-63, MC3T3-E1 *E. coli*, *S. aureus*	The laser-induced thermal ablation of BP synergizes with tumor agents to improve the efficiency of tumor ablation. Meanwhile, the ROS released by BP can be efficiently antibacterial	He et al. (2022a)
		*In vivo*: UMR-106 subcutaneous OS of male BALB/c mice, distal femoral condyle defect models of SD male rats		
Chemotherapy combined with PTT and PDT	A multifunctional gelatin methacrylate/DA methacrylate adhesive hydrogel coating containing BP nanoparticles protected by polydopamine	*In vitro*: HELA, MC3T3-E1 *E. coli*, *S. aureus*, HUVEC	Antitumor, antibacterial and bone regeneration	Li et al. (2023d)
		*In vivo*: HELA in left axilla of nude mouse, femoral condyle defect models of SD male rats		

#### 7.1.1 PTT

PTT, in which light energy is converted into heat energy to ablate the lesion by means of a PTA, has been widely used in tumor treatment (Lv et al., 2021). When the temperature in the localized area of the tumor rises to 41°C–48°C, it results in the death of the tumor cells. In 1995, Chen et al. first found that indocyanine green combined with an 808 nm diode laser produced a photothermal effectiveness that could kill breast tumor cells (Chen et al., 1995). In 2014, Liu et al. first reported that PTT effectively inhibited the growth of OS *in vivo*. Subsequently, a large number of studies on the *in vivo* and *in vitro* applications of PTT in OS had been published since 2014 (Liu et al., 2014c). Two key elements of the PTT, the photothermal agent and the light source. Different types of photothermal agent had been reported, including organic-based materials, carbon-based materials, metal-based materials (Sun et al., 2021). However, due to the limitations of the previous photothermal agent, sometimes heat inevitably leaks out of the target tissue and damages the surrounding normal tissue. Therefore, with the tremendous advances in photothermal agent modification and nanosizing, a number of nanoparticles are continuously being investigated as the most promising photothermal agents for current research.

**FIGURE 7:**
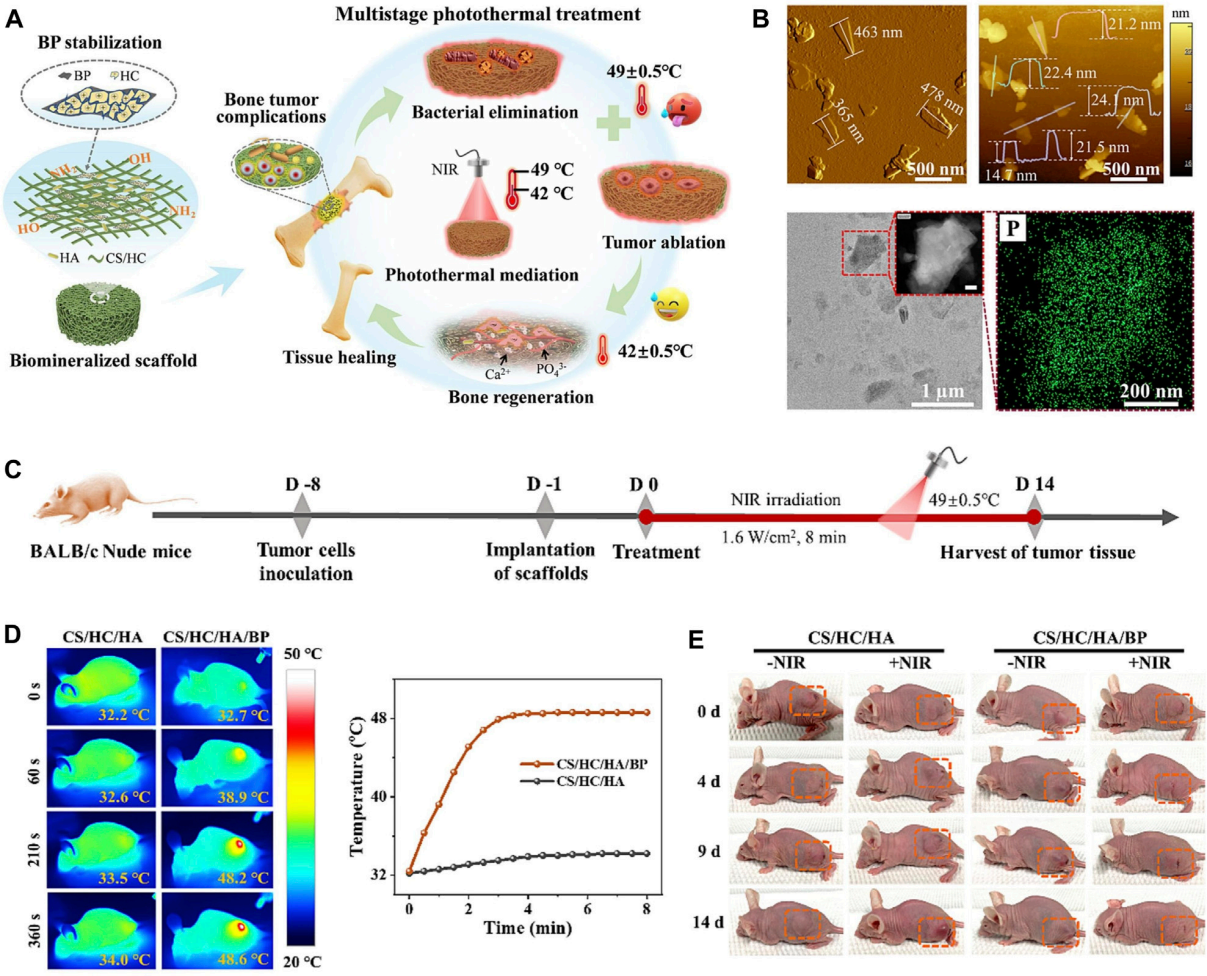
**(A)** Biomineralized CS/HC/HA/BP scaffold with bone-like hierarchical structure for multistage photothermal treatment of the severe complications associated with bone tumors: bacterial elimination, tumor ablation and subsequent osteogenesis. **(B)** AFM topographic images, TEM image and STEM-EDX elemental mapping images of BP sheets. **(C)** Schematic design for the model establishment and photothermal treatment process of the solid tumor in nude mice. **(D)** The corresponding infrared thermographic maps and time-dependent temperature curve of the tumor-bearing nude mice implanted with CS/HC/HA and CS/HC/HA/BP scaffolds irradiated with NIR laser. **(E)** Typical photographs of the tumor-bearing mice with or without NIR irradiation. (Copyright (Zhao et al., 2023b)).

BP, because of its excellent photothermal conversion efficiency has been used in studies of breast tumor and hepatoma (Chen et al., 2022b; Xie et al., 2023), while there was no review of the photothermal effectiveness of BP about OS. OS, which are often deep and large bone defects that remain after tumor resection, are well suited for NIR-assisted PTT with BP nanoparticles. To achieve the vision of tumor radio-fusion elimination and bone regeneration, Yang et al. first incorporated BPNSs into a 3D printed BG scaffold to treat mice with OS. Under NIR, the temperature of the BG scaffolds increased from 32.4°C to 68.7°C with 808 nm laser irradiation for 5 min (BPNSs concentration = 200 ppm, power density = 1.0 W cm^-2^), and the photothermal effectiveness did not deteriorate with increasing temperature. *In vivo*, the temperature of mice OS tissue increased from 30 °C to 55 °C within 1 min and to 58 °C within 5 min. After 14 days, the tumors were completely eliminated without recurrence (Yang et al., 2018). Bacterial infections may also occur following the removal of the OS. Previous studies have shown that long chains of cationic polymers can penetrate the cell membranes of microorganisms, inducing ion leakage and cell death. Negatively charged BP nanoparticles can easily adsorb positively charged macromolecules via electrostatic interactions. Hydroxypropyl-trimethyl ammonium chloride CS (HC) has been shown to improve the stability of BP in physiological environments and to achieve acceptable antibacterial efficacy at 56 °C. This means that BP-based composites can also be antibacterial during the photothermal conversion period. Specifically, Zhao et al. (Zhao et al., 2023b) incorporated BPNSs into a CS/HC matrix mineralized with nano-hydroxyapatite (HA) to prepare CS/HC/HA/BPNSs scaffolds with bone-like hierarchical structure (Figure 7). *In vitro* and *in vivo* validation demonstrated that antibacterial and antitumor could be achieved in a one step at 49°C ± 0.5°C due to synergistic effectiveness of HC and thermotherapy; at a temperature of 42°C ± 0.5°C, NIR promotes subsequent osteogenesis. In addition to BPNSs, BPQDs are also used in PTT of tumors. However, the instability of BPQDs is a barrier to their clinical application. After interaction with oxygen, light, and water, BPQDs are susceptible to oxidation and degradation to produce phosphates, leading to a decrease in the efficiency of photothermal conversion and thus in the efficacy of therapy. To overcome this difficulty, Hu et al. (Hu et al., 2022a) encapsulated BPQDs in PLGA nanospheres and integrated them into WW/RSF hydrogel scaffolds (BP/WW/RSF) for tumor abatement and bone regeneration. The results of *in vitro* photothermal conversion efficiency experiments demonstrated that a power density of 1.0 W cm^-2^ was sufficient to induce a temperature increase of 27.2°C. *In vivo*, under 1 W cm^-2^ irradiation, the temperature of the tumors increased rapidly from 30 °C to 51.9°C in 5 min, and the tumors were effectively controlled over a 4 weeks observation period. Compared to other treatments, such as combining a carrier with a therapeutically toxic chemotherapeutic agent, PTT is able to mitigate unwanted side effects during surgical implantation because the external NIR irradiation after implantation of BP-based composites is much less harmful.

#### 7.1.2 Chemical-photothermal therapy (CPT)

Treatment of OS usually involves surgical removal of the tumor tissue. However, it is difficult to ensure that the mass is completely removed through surgery, and residual OS cells can cause the tumor to recur. To prevent tumor recurrence, postoperative adjuvant chemotherapy has become the standard treatment strategy for OS (Hu et al., 2022b). However, the side effects and adverse reactions of systemic chemotherapy, such as alopecia, weight loss, and bone marrow suppression, cause severe adverse emotional responses in patients. Although studies have shown that local chemotherapy can do a good job of removing tumor cells and inhibiting recurrence and avoiding systemic medication, subsequent tissue regeneration was hampered by the side effects of locally released high doses of chemotherapeutic agents (Dai et al., 2020). Therefore, finding an efficient treatment option for OS that can reduce the chemotherapy dose is of great clinical application.

**FIGURE 8:**
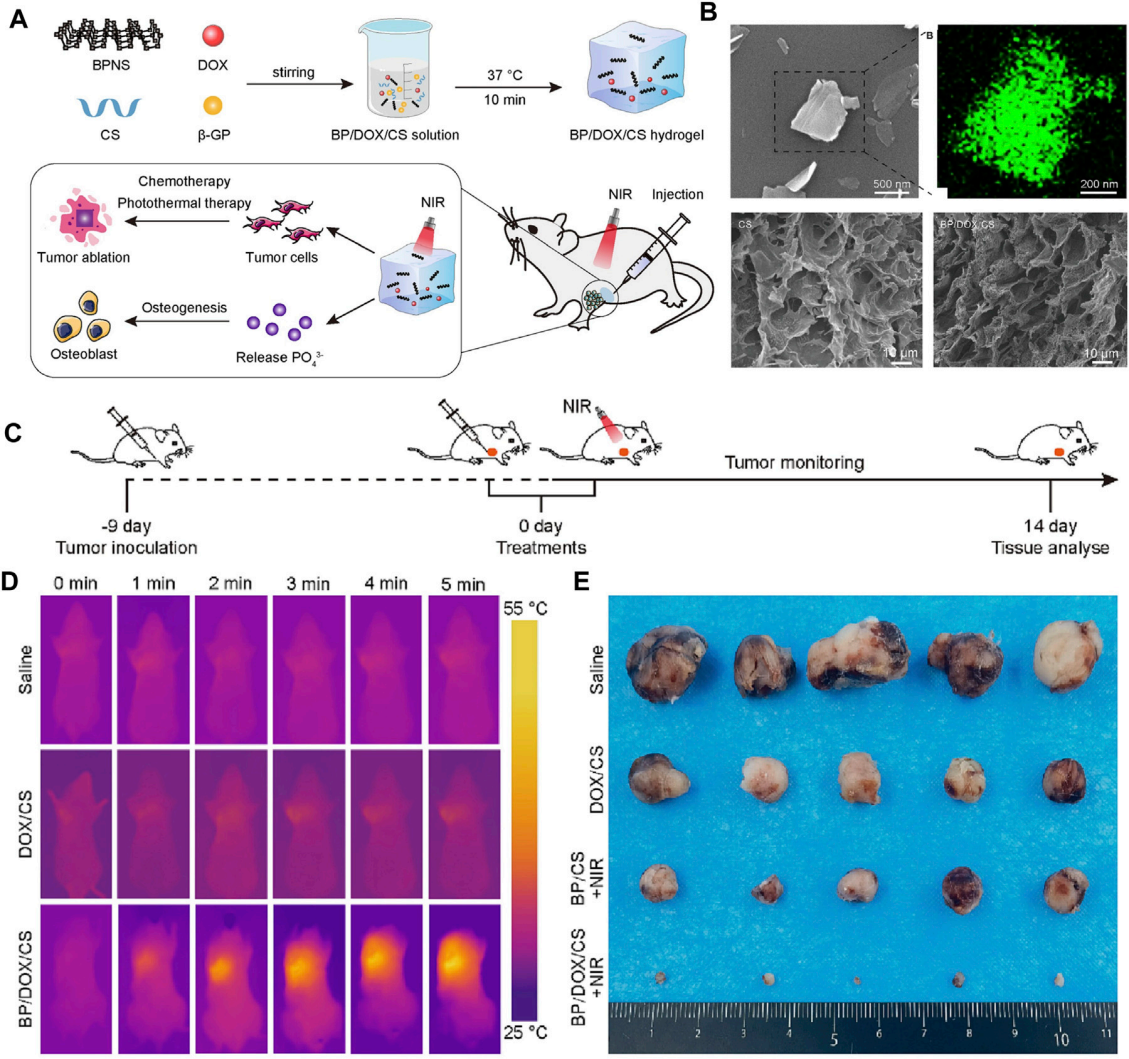
**(A)** Schematic illustration of the injectable BP/DOX/CS hydrogel for synergistic photothermal-chemotherapy bone tumor and enhancing osteogenesis. **(B)** SEM image and EDS energy spectrum analysis of BPNS, SEM images of CS hydrogel and BP/DOX/CS hydrogel. **(C)** Treatment schedule. **(D)** Thermographic images of mice exposed to 808 nm NIR light for 5 min. **(E)** Photograph of dissected tumor samples of different treatment groups at 14 days (Copyright (Li et al., 2023c)).

BP nanoparticles have a high surface area-to-volume ratio and can be well loaded with chemotherapeutic agents such as DOX and gemcitabine. In addition, the photothermal effectiveness reduces the content of chemotherapeutic agents to minimize the local chemotherapeutic side effects, making PTT in combination with chemotherapy a promising therapeutic strategy. In 2020, the combined chemotherapy with PTT using BPNSs was firstly reported for OS therapy (Wang et al., 2020b). In this report, the release of DOX by BPNSs due to the presence of photothermal effectiveness had an accelerated release behavior at 10 min from the beginning of the release, reaching a 2-fold instantaneous release of 11% ± 1.8% within 24 h. Moreover, tumor recurrence was significant in the PTT group compared to the PTT combined with chemotherapy group. This suggests that PTT and chemotherapy can play a synergistic role in OS therapy. Based on the above theoretical foundation, later, Ma et al. designed a multifunctional photothermal synergistic chemotherapeutic platform for OS therapy (Fs-BP-DOX@PDA) that integrates antibacterial and bone regeneration (Ma et al., 2022a). Shortly, femtosecond laser direct writing was firstly utilized to prepare slot micro-nanostructures (Fs-NiTi), then BPNSs were modified onto Fs-NiTi to construct multiscale hierarchical structure (Fs-BPNSs), and then PDA was used to modify Fs-BPNSs, and DOX was loaded onto Fs-BPNSs by electrostatic adsorption and an efficient cyclic loading method. Results showed that Fs-BPNSs were 93.2% loading efficient on DOX, and that Fs-BPNSs-DOX@PDA had good biocompatibility and synergistic antitumor efficacy with photothermal chemotherapy. Recently, an injectable hydrogel for OS therapy and bone regeneration was designed by encapsulating BPNSs and DOX in a CS-based hydrogel (BPNSs/DOX/CS) (Figure 8) (Li et al., 2023b; Li et al., 2023c). Antitumor effect *in vitro* indicated that chemotherapy and PTT alone had limited inhibitory effects on the tumors, while the BP/DOX/CS group had almost no cancer cells remained, indicating the antitumor advantage of Chemo-PTT combined treatment. In addition, for chemotherapy or PTT alone, tumor volume was significantly increased, although DOX/CS and BP/CS + NIR treatments suppressed tumor growth to some extent. In contrast, tumor growth was significantly delayed in BP/DOX/CS + NIR-treated mice, and the tumors disappeared after 14 d. This also indicated that BP/DOX/CS effectively inhibited tumor growth by combined photothermal chemotherapy. The low photothermal performance of BP in the NIR-II biological window (1,000–1,500 nm) limits its application in biomedical fields. In 2024, Wu et al. (Wu et al., 2024a) modified BP by lattice reconfiguration and designed a nanocomplex based on BPQDs, where the rate and the amount of DOX released are controlled by the light intensity and the irradiation time of a 1,064 nm laser irradiation. *In vivo* experiments have shown that nanomedicine has excellent tumor targeting capability and biocompatibility, and can achieve complete tumor ablation with a combination of PTT and chemotherapy.

In the above sections, we reviewed the excellent results of the photothermal effectiveness on BPQDs for OS therapy. In addition, as drug carriers with negatively charged surfaces, BPQDs can have better drug-carrying properties by electrostatically adsorbing multiple agents with positively charged surfaces. It is a good idea to combine the photothermal effectiveness of BPQDs with the drug-carrying nature of OS therapy. However, BPQDs are known to be susceptible to oxidative degradation. To accomplish this idea, Xu et al. combined BPQDs with a biofilm to load DOX. Specifically, BPQDs were loaded with DOX firstly, and then OPM was constructed by fusing OCM with PM simultaneously. Finally, OPMs were used to encapsulated the BPQDs-DOX surface. The BPQDs-DOX@OPM DDS has high biocompatibility, targeting and photothermal chemotherapeutic effectiveness (Xu et al., 2023). In contrast to BPQDs-DOX, BPQDs-DOX@OPM showed significant clustering in tumor tissue 24 h after intravenous administration and did not show significant reduction after 48 h. After 72 h of irradiation with 808 nm NIR light, the DOX release rate in the BPQDs-DOX@OPM group was 76.04%, which was significantly higher than that in the non-lighted group. At the same time, the cell proliferation activity of the BPQDs-DOX@OPM group was maintained at more than 95%. The inhibition of tumor growth rate was more pronounced in the BPQDs-DOX@OPM group, suggesting that PTT in combination with chemotherapy has a more significant advantage over single-agent therapy, with OS therapy having a more pronounced advantage.

Thermal generation during PTT can either accelerate the release of chemotherapeutic agents or directly kill tumor cells. At the same time, ROS released during PTT can inhibit drug efflux P-glycoprotein pumps in multidrug-resistant tumor cells and enhance chemotherapeutic efficacy. Chemotherapeutic agents, in turn, can ameliorate the limitations of light penetration in PTT and increase the sensitivity of tumor cells to hyperthermia or ROS. Combining these two therapies can have a synergistic effect, increasing tumor killing efficiency, reducing tumor recurrence rate, and reducing the dose of subsequent chemotherapeutic agents used, thereby mitigating the side effects of chemotherapy. The photothermal chemotherapy of BP nanoparticles can also promote bone regeneration and produce bactericidal potency.

#### 7.1.3 PDT

In addition to its photothermal nature, BP nanoparticles have photodynamic properties that allow them to form PDT. Three basic elements of PDT, namely, photosensitizer, light source, and ROS, are used to kill tumor cells. The specific mechanism of PDT is that, in the presence of light of a specific wavelength, photosensitizer is activated from the ground state to an excited state, and the activated PS is able to react directly with a substrate (cellular membrane or a molecule) to form free radicals and interact with oxygen to generate ROS (type I reaction) or directly transfer energy to oxygen to form a single linear state of oxygen (^1^O_2_), which directly passes through ^1^O_2_ to cause cellular damage (type II reaction). In short, light energy is converted into chemical energy in an appropriate manner. The fabrication of photosensitizers, the design of PDT strategies and the application of PDT for bone tumor therapy were described comprehensively in a recent review by Xie et al. (Xie et al., 2021) Most of the research on the use of PDT with BP nanoparticles for tumor therapy has focused on breast cancer, lung cancer, and cervical cancer. Whereas there are few studies on PDT with BP nanoparticles for OS, in this section we would like to summarize the effectiveness of PDT with BP nanoparticles for OS therapy.

In 2005, Katsuyuki et al. (Kusuzaki et al., 2005) firstly reported a clinical study of PDT for OS therapy in which they surgically excised malignant bone tumor lesions in 10 patients before topically administering a 1 μg/mL acridine orange (AO) solution. The blue light was then irradiated for 10 min. All patients (AO-PDT: five patients, AO-PDT combined with 5-Gy radiotherapy: five patients) survived. At a follow-up of 24–48 months, there was no tumor recurrence in the five patients who received AO-PDT combined with radiotherapy, and one tumor recurrence in the five patients who received AO-PDT. Later, it was shown that PDT mediated by amino ketoglutarate can cause *in vitro* death of human OS cells MG-64. Meanwhile, Yu et al. (Yu et al., 2017) summarized seven feasible improvements of PDT for OS therapy, including further exploration of photosensitizer and light sources, as well as combination with other therapeutic techniques such as targeted therapy and immunotherapy. Although PDT has gradually become a hot spot in tumor treatment research, the related research of OS is still insufficient, and photosensitizer is limited to a dozen types, including acridine orange, 5-methylene blue, aminolevulinic acid (ALA), HiPorfin, 5, 15-bis (2-bromo-5-hydroxyphenyl) porphyrin, aloe emodin, Foscan and Foslip, pyrophospho-A methyl ester (MPPa), zinc phthalocyanine-Bovine Serum albumin (ZnPc/BSA) nanoparticles, *etc.* However, the rapid development of nanoparticles has provided a major breakthrough for PDT in tumor therapy for photosensitizer flaws, including deep tumors such as OS. The modification of nanoparticles and the enhanced permeation retention (EPR) effectiveness featured by nanoparticles reduces the deposition and cytotoxicity of photosensitizer in the liver, kidneys, and other normal organs and tissues, which enhances the promising application of PDT in OS therapy. Based on the excellent photodynamic effectiveness, PDT with BP nanoparticles has been widely used for sterilization (Ding et al., 2021) and tumor treatment (Gnanasekar et al., 2023). In 2019, Raucci et al. found that PDT at 2D BP nanoparticles inhibited the metabolic activity of OS cells (SAOS-2) by increasing the production of anti-inflammatory cytokines (interleukin-10) and inhibiting the synthesis of pro-inflammatory mediators (interleukin-6) through *in vitro* modeling, while inducing the proliferation and osteogenic differentiation of human preosteoclasts and human mesenchymal stem cells (Raucci et al., 2019a). Therefore, BP nanoparticles are indeed one of the promising photosensitizer candidates for OS therapy. However, efficient and precise delivery of photosensitizer into tumor cells and subcellular organelles remains a challenge due to the step-reduction delivery dilemma (SRDD). Recently, Zhang et al. (Zhang et al., 2022c) designed a cascade-targeted NIR II fluorescent nanoparticle (NPER/BO-PDT), which can be targeted to delivery photosensitizer into endoplasmic reticulum (ER) subcellular organelles. NPER/BO-PDT under NIR light irradiation generates sustained ROS in the endoplasmic reticulum, which results in sustained endoplasmic reticulum stress and induces a potent immunogenic cell death (ICD) that can promote immune recognition, overcoming SRDD, to achieve synergistic killing of OS cells and achieve more effective so-called photodynamic immunotherapy. This provides ideas for improving the effectiveness of PDT with BP nanoparticles. In conclusion, PDT of BP nanoparticles in OS therapy is a promising direction, but the research is still in the experimental stage in tumor models and clinical applications are still very limited. Therefore, more comprehensive and further studies in the field of PDT are needed.

#### 7.1.4 Chemotherapy combined with PTT and PDT

**FIGURE 9:**
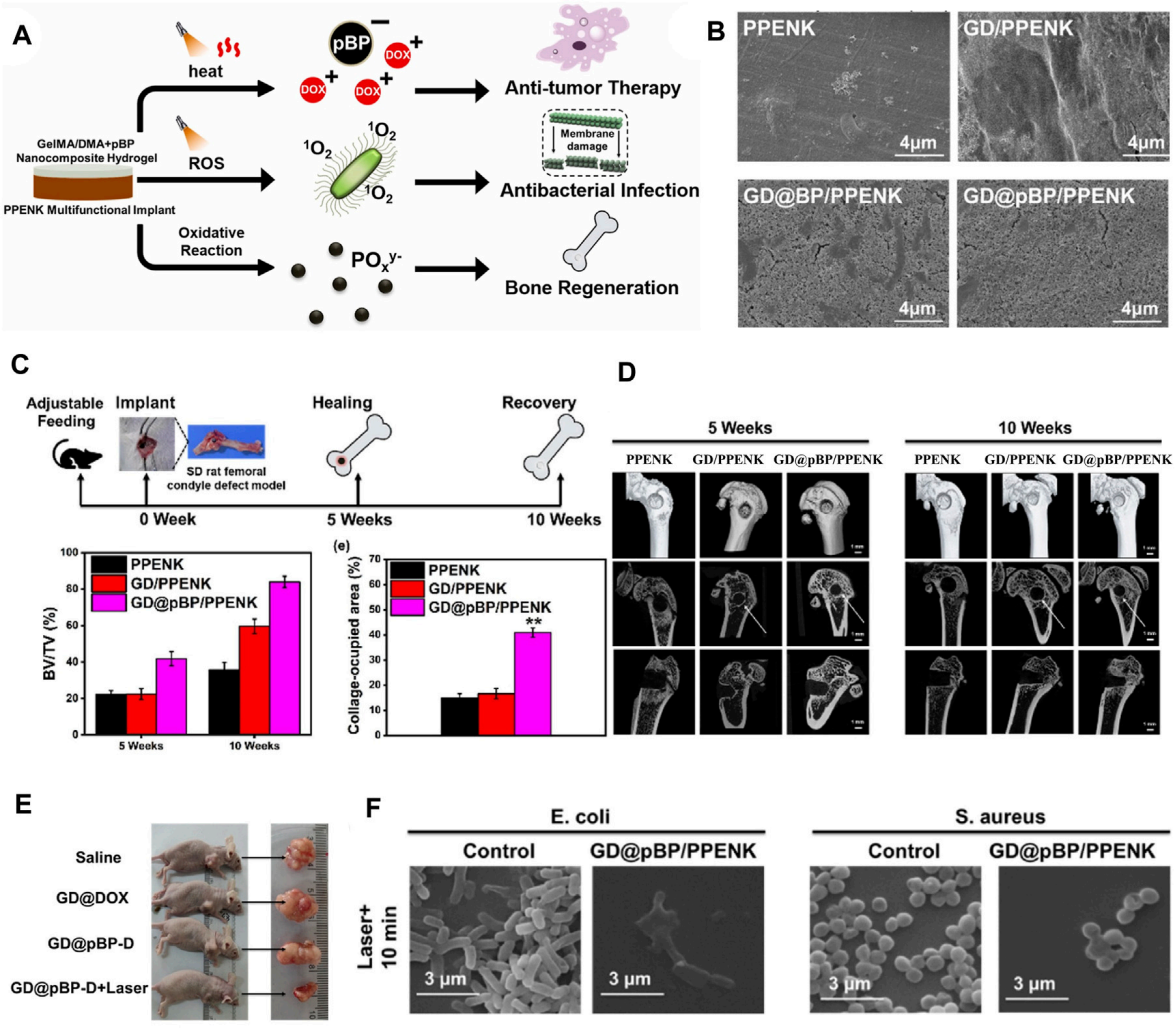
**(A)** The graphical abstract of synergistic photothermal-chemotherapy and photodynamic therapy for bone tumor therapy, enhancing osteogenesis and kill bacteria. **(B)** SEM morphology images of PPENK, GD/PPENK, GD@BP/PPENK and GD@pBP/PPENK after mineralization in SBF with 14 days **(C)** Overall timeline of bone healing and quantitative analysis of bone volume to tissue volume (BV/TV) at 5 weeks and 10 weeks, n = 3 for each group. **(D)** Micro-CT images of femur defects showed different bone regeneration at 5 weeks and 10 weeks **(E)** Images of representative tumor in mice with different treatments. **(F)** Typical morphology of *E. coli*. and *S. aureus* incubated with GD@pBP/PPENK after laser irradiation. (Copyright (Li et al., 2023d)).

In the clinic, there is a need to face the practical issue of repairing large bone defects after OS resection, as well as post-operative chemotherapy to kill residual tumor cells and prevent bacterial infections during incision healing. In recent years, osteogenic scaffolds capable of providing PTT and PDT capabilities have received increasing attention from researchers. These scaffolds promote bone regeneration and kill bone tumor cells via PTT/PDT. For example, some research groups have developed nanocomposite tissue scaffolds by incorporating HA, calcium silicate (CaSiO_3_)) and photo thermite (polydopamine, strontium ferrite (SrFe_12_O_19_), and graphene oxide (GO)) into degradable polymers (CS or sericin proteins), which enable bone regeneration and killing of tumor cells (Ma et al., 2020; Yao et al., 2021). However, the weight-bearing capacity was insufficient. Recently, 3D-printed titanium-based bone scaffolds have also been developed with photothermal and antibacterial properties that kill bone tumor cells and have an antibacterial effectiveness (Zhang et al., 2022a). However, the high modulus of elasticity of titanium can lead to stress shielding. BPNSs have a graphene-like 2D nanostructure and are highly biocompatible. The material is negatively charged and can bind positively charged drugs to deliver anti-tumor agents. It can thermally ablate tumor cells under NIR and release ROS during degradation, demonstrating strong PDT efficacy, and the degradation product PO_4_^3-^ can promote bone regeneration *in vivo*. BP nanoparticles are already widely used in biomedicine. BP nanoparticles have been widely used in biomedical applications. However, the utility of BP nanoparticles for OS therapy based on photothermal efficacy, photodynamic efficacy, and drug delivery properties has been less explored. Recently, He et al. prepared a BPNSs/CS composite coating and deposited it on a 3D printed PEEK bone scaffold. The PEEK scaffold provides mechanical properties and structure similar to those of natural bone, and the composite coating allows simultaneous chemotherapy via laser-induced thermal ablation of tumor cells and pH-sensitive tumor drug release. BPNSs also released ROS to effectively eradication of *E. coli* and *Staphylococcus aureus*, and BPNSs degradation products promoted bone regeneration (He et al., 2022a). However, due to the difficulty of processing the material and its low solubility, which leads to high costs, there is an urgent need to find alternatives to PEEK. PPENK represents a new type of high-performance resin that has mechanical strength similar to that of natural human bone and is easier to process than PEEK, making it an ideal candidate for bone implant materials. Under 808 nm laser light, BPNSs can produce ROS to eliminate bacterial infections. During the slow degradation process, BPNSs not only efficiently consume excess ROS to avoid ROS-induced apoptosis in normal cells, but also degrade into PO_4_^3-^ to promote osteogenesis. Li et al. (Li et al., 2023d) developed a BP-based hydrogel coating to modify the surface of PPENK for OS therapy. This versatile gelatine methacrylate/dopamine methacrylate adhesion hydrogel coating containing PDA-protected BP nanoparticles prepared by photo-cross-linking is able to work synergistically with BPNSs whose formal efficacy controls the release of Adriamycin hydrochloride. Meanwhile, BPNSs can generate ROS under 808 nm laser light to eliminate bacterial infection. During the slow degradation process, BPNSs not only effectively consumed excess ROS to avoid ROS-induced apoptosis in normal cells, but also degraded into PO_4_^3-^ to promote osteogenesis (Figure 9). In summary, BP nanocomposites offer a promising clinical strategy for treating patients with OS by combining chemotherapy with PTT and PDT to achieve antitumor, antibacterial and bone regeneration efficacy.

### 7.2 BP nanoparticles for bone regeneration

OS therapy often results in severe bone defects that require tissue-engineered scaffolds for bone regeneration, since critical-sized bone defects cannot self-heal. The healing of bone defects secondary to tumor resection is hampered by the proliferation of residual tumor cells. At the same time, it has been shown that high doses of chemotherapeutic agents at OS lesions affect bone regeneration (Zhou et al., 2017). Therefore, there is significant clinical value in designing a versatile therapeutic platform that completely removes residual bone tumor cells and effectively accelerates bone regeneration.

BP nanoparticles can be broken down into toxic-free phosphates, which can promote bone regeneration by stimulating the migration of calcium and phosphorus ions and increasing the expression levels of osteogenesis-related proteins. It has been shown that PO_4_^3-^ degraded from BPNSs in a physiological condition promotes the proliferation and differentiation of preosteoclasts, and that BPNSs improves the biocompatibility of the composite (Xu et al., 2021a). Xu et al. (Xu et al., 2021b) explored the bioactive mechanism of BPNSs, which were oxidized into oxidized BPNSs with bisphosphonate-like phosphate groups. This phosphate group not only acts as a binding site for calcium ions, but also inhibits bone resorption by inhibiting the phosphorylation of p38 (MAPK) in the osteoclast precursor-kB signaling pathway and down-regulating the expression of NFATc1. Long et al. (Long et al., 2023) explored the mechanism of immunomodulation of bone by BP nanoparticles, and the PLGA/BP scaffolds were able to inhibit inflammation by stimulating macrophage M2 polarization, and promote osteogenic differentiation of BMSCs through activation of the PI3K-AKT signaling pathway. Under NIR, the photothermal properties of the BP nanoparticles promoted auto-degradation, and the photothermal effectiveness facilitated the osteogenic differentiation of BMSCs by promoting the expression of HSPs. Tan et al. (Tan et al., 2022) introduced BPNSs encapsulated in BMSCs membranes into CS/collagen composite hydrogels and found that BPNSs could stimulate osteoblast recruitment through activation of heat shock protein-mediated matrix metalloproteinases and the ERK-WNT/β-Catenin-RUNX2 axis under NIR.

**FIGURE 10:**
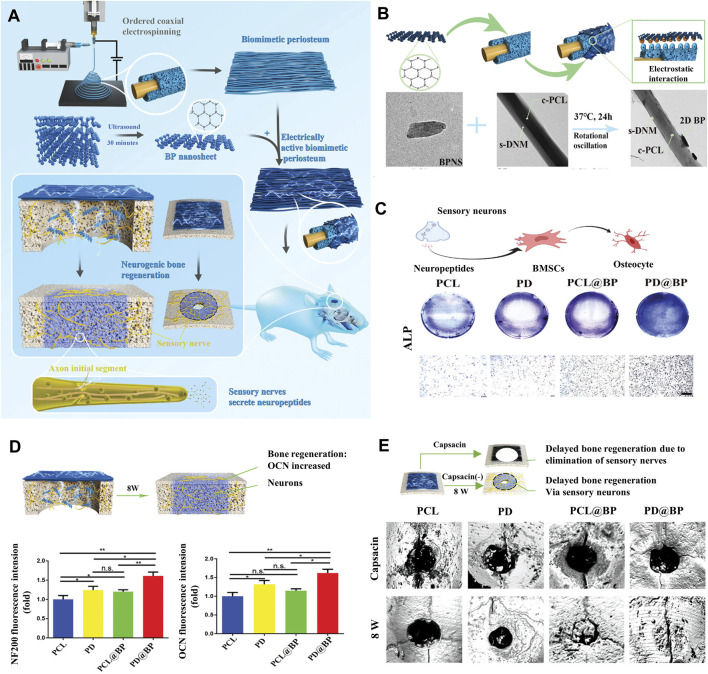
Strategies for BP nanocomposites for bone regeneration. **(A)** Schematic diagram of the PD@BP promoting neurogenic bone regeneration. **(B)** Characterization and properties of electrically active electrospinning periosteum. **(C)** Electrically active periosteum promotes the osteogenic transformation of BMSCs through sensory nerves *in vitro*. **(D)** Diagram of nerve-induced osteogenic repair after 8 weeks and the corresponding quantitative analysis of skull defect area. **(E)** Detection of electrically active periosteal osteogenesis with micro-CT *in vivo*. (Copyright (Su et al., 2023)).

Bone regeneration that includes nerve and blood vessel rebuilding is more consistent with natural tissue. In order to realize vascularized bone regeneration, Wang et al. (Wang et al., 2021) used 3D printing to prepare photo-thermo-responsive channel scaffolds. The BPNSs enabled the scaffolds to have reproducible contraction and expansion properties under NIR, which facilitated the growth of blood vessels in the scaffold orifices during the process of bone regeneration. Recently, Miao et al. developed a BPNSs-based dynamic DNA hydrogel and integrated it with 3D-printed polycaprolactone (PCL) bone scaffolds to construct a bioactive hydrogel -scaffold composite structure, which enables sustained release of vascular endothelial growth factor (VEGF) due to non-covalent interactions between VEGF and BPNSs (Miao et al., 2023). In order to achieve neurogenic bone regeneration, Jing et al. (Jing et al., 2023) prepared a photosensitive hydrogel with electric conduction by incorporating magnesium-modified BPNSs (BPNSs@mg) into gelatin methacrylate (GelMA). The nano-sheets with electric conduction released by BPNSs@mg and bioactive ions synergistically promote the migration and secretion of Schwann cell, which contributes to neural axon growth, and provide a new idea for neurogenic bone regeneration. Su et al. (Su et al., 2023) prepared a biomimetic periosteum using ordered coaxial electrospinning, and constructed an electrically active biomimetic periosteum by loading BPNSs onto the biomimetic periosteum via electrostatic action (Figure 10). In this study, BP with excellent electrical conductivity was the main trigger to stimulate the efflux of nerve vesicles, then the PD@BP biomimetic periosteum mainly induces neurogenesis and osteogenesis through the Fanconi anemia pathway, providing a new strategy for bone regeneration. To achieve neuro-vascularized bone regeneration, Xu et al. (Xu et al., 2022a) designed a bilayer hydrogel with a top layer consisting of GelMA hydrogel with BPNSs@mg and a bottom layer consisting of two interpenetrating polymer networks of GelMA, polyethylene glycol (PEG) and β-TCP nanocrystals. *In vivo* and *in vitro* experiments demonstrated that the top layer hydrogel significantly promoted angiogenesis by inducing endothelial cell migration and facilitated the expression of neural-related proteins in neural stem cells (NSCs), and the bottom layer hydrogel promoted osteogenic differentiation of BMSCs. The above work has positive implications for the design of biomaterials for neuro-vascularized bone regeneration.

Wear and tear of bone tissue engineering materials in the body may lead to inflammatory reactions and bacterial infections. To cope with this problem, Zhao et al. (Zhao et al., 2022a) designed a novel CS/PCL/BP/PDA@Ag scaffold inspired by chloroplasts, with the BP resembling the cyst-like membrane and matrix layer in chloroplasts, integrated with a network of CS and polycaprolactone fibers. The results of *in vivo* and *in vitro* experiments showed that the scaffold had significant photothermal osteogenesis and anti-infection effectiveness. Li et al. (Li et al., 2022) prepared bone morphogenetic protein-2 (BMP-2) PLGA microspheres by the complex emulsion method and then coated them on a scaffold composed of a mixture of BPNSs and PLGA to form a BMP-2@BPNSs scaffold. The BMP-2@BPNSs scaffold promotes osteogenic differentiation through the release of BMP-2 and upregulates the expression of heat shock proteins under NIR. In addition, BPNSs have shown antibacterial efficacy under NIR to prevent clinical infections. Wu et al. (Wu et al., 2021) coordinated BPNSs with a zinc sulfonate ligand (ZnL_2_) and integrated it onto the surface of the HA scaffold. BPNSs may kill bacteria under NIR, ZnL_2_ exerts pressure on the bacterial cell membrane and disrupts the cell membrane. More importantly, degradation products of ZnL_2_ and PO_4_^3-^ composite scaffolds have a positive effect on osteogenic differentiation.

In OS therapy, it is necessary to fill the scaffold in order to achieve bone regeneration. The scaffold for bone regeneration after OS resection include PEEK scaffold (He et al., 2022a), PPENK scaffold (Li et al., 2023d), hydrogel bionic scaffold) (Hu et al., 2022a), BG scaffold (Yang et al., 2018), nanocomposite scaffolds (Wang et al., 2020a). A customized scaffold for bone regeneration using 3D printing with BP complex as a coating improved the antitumor, bone regeneration, and antibacterial effectiveness of the scaffold. He et al. (He et al., 2022a) designed a BPNSs/CS composite coating to modify 3D-printed PEEK scaffolds for OS therapy, bone repair, and antibacterial. Composite coatings could potentially be used for OS therapy through on-demand laser-induced heating and pH-sensitive release of chemotherapeutic agents, while ROS released by BPNSs could kill bacteria in response to potential post-operative infections. In addition to being used as a coating for scaffolds, BP nanoparticles can be directly synthesized into hydrogels that can be injected into lesions for effective tumor eradication and bone tissue reconstruction. Li et al. (Li et al., 2023c) designed an injectable multifunctional hydrogel (BPNSs/DOX/CS) for synergistic photothermal chemotherapy and bone regeneration, in which BPNSs and DOX were encapsulated in an injectable CS-based hydrogel. The BPNSs/DOX/CS hydrogels exhibit good photothermal efficacy at NIR and good drug delivery capacity for sustained DOX release. In summary, BP nanoparticles are competitive candidates for vascularized and even neovascularized bone regeneration by participating in the expression and degradation of osteogenesis-related proteins into PO_4_^3-^. Moreover, the formal properties of BP nanoparticles can accelerate their own degradation and biomineralization, stimulate osteogenic differentiation, and also be used for OS photothermal therapy.

### 7.3 BP nanoparticles for antibacterial

Treatment of OS often requires the application of implants, and surgical-related bacterial infections reduce the usefulness of implant materials in the body. Therefore, the optimal implant material for OS therapy should not only satisfy the need to kill tumor cells and repair bone defects, but also have the property to avoid bacterial infection. Fortunately, in addition to the characteristics of BP nanoparticles that not only kill tumor cells and osteoblasts as described above in this review, BP nanoparticles also have antibacterial bio-activities without the problem of drug resistance (Liu et al., 2020).

**FIGURE 11:**
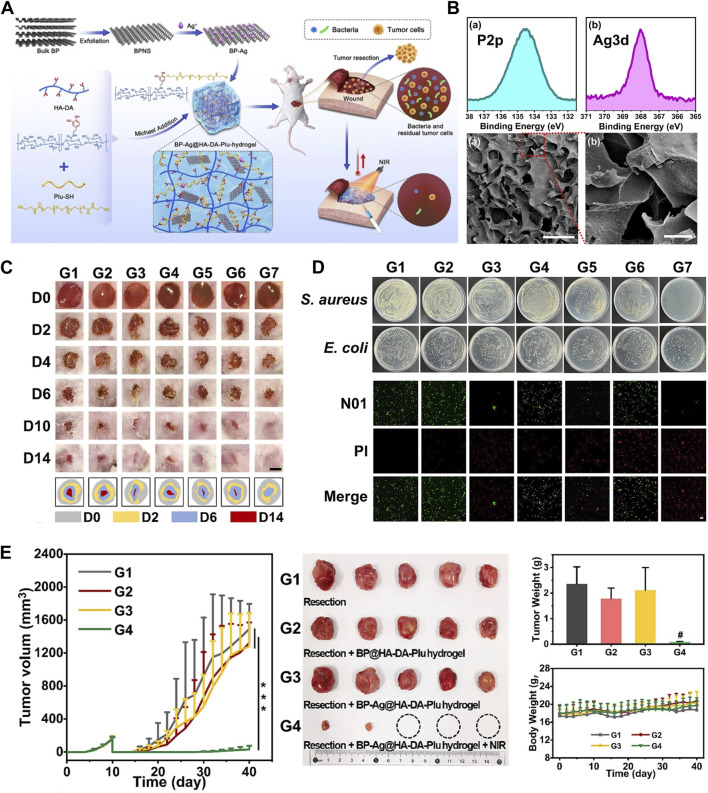
Strategies for BP nanocomposites for antibacterial. **(A)** Schematic illustration of the fabrication and treatment process of the BP-Ag@HA-DA-Plu hydrogel. **(B)** XPS P2p and Ag3d spectra of BP-Ag and the SEM image of BP-Ag@HA-DA-Plu hydrogel. **(C)** Photographs of the healing process of S. aureus-infected wound after different treatments. **(D)** Photographs of *S. aureus* and *E. coli* colonies after different treatments. **(E)** Tumor growth curves, weights of excised tumors, bodyweight change curves and photographs of excised tumors of mice with different treatments (Copyright (Li et al., 2023e)).

Earlier, researchers found that BPNSs produce ROS when irradiated with visible light, which acts as an antibacterial agent. In order to promote ROS generation to enhance the bactericidal properties, several BP nanocomposites have been developed, such as BP/Ag nanocomposites (Liang et al., 2020; Li et al., 2023e), BP/Au nanocomposites (Aksoy et al., 2020), BP/Au-ZnO nanocomposites (Naskar et al., 2021), BP/ZnO nanocomposites (Fang et al., 2022) and BP/Cu nanocomposites (Zhang et al., 2020b). In addition to inducing ROS generation to kill bacteria (Aksoy et al., 2020; Li et al., 2023d), photothermal conversion is another important mechanism for the antibacterial properties of BP nanoparticles (Zhao et al., 2023b). Li et al. developed a postoperative BP-Ag nanocomposites-loaded dopamine-modified hyaluronic acid-Pluronic^®^ F127 (BP-Ag@HA-DA-Plu) hydrogel for simultaneous prevention of tumor recurrence and wound infection (Figure 11). (Li et al., 2023e) This study demonstrated that utilizing the synergistic broad-spectrum antibacterial ability of silver nanoparticles and the photothermal effect of BPNSs, the BP-Ag@HA-DA-Plu hydrogel inhibited *Staphylococcus aureus* and *Escherichia coli* better than the control. Also, BP-Ag@HA-DA-Plu hydrogel showed better healing of infected wounds and lower tumor recurrence. BP nanoparticles can also kill bacteria by disrupting their cell membranes by sharp edges. In addition to the above mechanisms, BP nanoparticles can manifest antibacterial activity through a variety of other mechanisms, such as interruption of bacterial respiration through electron transfer activity, magnetically assisted penetration, encapsulation and isolation, acid release, and reaction with essential nutrients (Zhang et al., 2020a). Even PTT combined with sonodynamic therapy (SDT) (Zeng et al., 2023), and PTT combined with PDT (Zhao et al., 2023a). However, BP nanoparticles readily react with water or oxygen adsorbed on the surface and are poorly stabilized in the environment, limiting their potential for applications. Surface coordination or chemical modification of BP can help to improve its stability, bacterial targeting ability, and antibacterial efficiency because of its photothermal/drug synergistic strategy. Currently, a variety of materials have been used to modify BPNSs to enhance antibacterial activity, including mannose (Zhao et al., 2023a), ε-poly-l-lysine (ε-PL) (Fu et al., 2022), CS (He et al., 2022a; Zhao et al., 2023b) and PDA (Li et al., 2023d; Zeng et al., 2023). Mannosylated BP nanosheets (Man-BPNSs) have been used to enable targeted photodynamic and photothermal combination therapy to kill bacteria (Zhao et al., 2023a). ε-PL can serve as an effective protector to avoid chemical degradation of bare BP, BP@ε-PL can kill multidrug resistant bacteria through membrane destruction, protein damage and DNA damage (Fu et al., 2022). He et al. prepared a BPNSs/CS composite hydrogel as a coating for 3D-printed PEEK bone scaffolds for OS therapy, and found that the antibacterial activity of the BPNSs/CS composite was superior to that of bare BP (He et al., 2022a). In conclusion, these strategies provide new ideas for enhancing of antibacterial efficacy of OS therapy.

## 8 Conclusion and future prospects

Research and development of OS therapy continues to drive large-scale, intense and long-term efforts worldwide. BP, as a 2D nanomaterial with excellent PDT/PTT properties, large specific surface area, folded structure, good biodegradability and high biocompatibility, has attracted much attention in recent years in the research of OS therapy. Their large specific surface area expands the possibility of drug loading, enabling more drug combinations. Meanwhile, they have good biocompatibility and biodegradability, making them ideal for biomedical applications. In summary, this work reviews the synthesis methods, physicochemical properties of BP nanoparticles and their unique biological properties such as biocompatibility, biodegradability, photothermal conversion ability, photodynamic effectiveness, drug delivery effectiveness, and also described the modification strategies of BP nanoparticles and the types of BP-based nanomaterials used in the OS therapy, and finally provides a comprehensive summary of the progress of the BP-based nanomaterials for OS therapy. There are some findings: 1) BPNSs are the most popular in many of the research groups covered in this review due to their physicochemical advantages; 2) PLGA- or CS-modified BPNSs account for the majority of the modified materials; 3) Electrostatic interactions are the main strategy for drug delivery and surface modification; 4) BP-based scaffolds are the predominant composite type for OS therapy; 5) Photothermal and photodynamic therapies are the main strategies for antitumor and antibacterial; 6) In contrast to other inorganic nanoparticles, BP nanoparticles have natural properties to promote bone regeneration.

The main approaches of BP-based nanomaterial in OS therapy include: 1) BP nanoparticles have excellent photothermal conversion effectiveness and can ablate tumor cells; 2) BP nanoparticles can generate cytotoxic ROS exhibiting antitumor properties effectively; 3) BP nanoparticles can be further classified into BPNSs and BPQDs, which are excellent nanoplatforms for drug delivery, and combined with PTT and PDT, can reduce the dosage of chemotherapeutic agents with decreased side effects. Considering that bone defect reconstruction is often required in OS therapy, prevention of implant-related bacterial infections is also an issue in the clinic. Fortunately, BP nanoparticles have outstanding properties for bone regeneration and antibacterial applications. The main strategies of BP nanoparticles in bone regeneration are: 1) Degradation of BP nanoparticles into phosphate captures calcium ions to form local mineralization and promote bone regeneration; 2) Utilizing the photo-thermal conversion ability of BP nanoparticles to activate the expression of osteogenic-related proteins such as heat shock proteins, alkaline phosphatase and other osteogenic proteins of local tissues to promote bone regeneration; 3) Utilizing the high surface area-to-volume ratio of BP nanoparticles to delivery bioactive ions and bio factors into the local microenvironment to improve the efficiency of bone regeneration. It can also act as an antibacterial, improving the local microenvironment and enhancing the efficiency of bone regeneration through formal and photodynamic efficiencies; 4) The high electrical conductivity of BP nanoparticles is beneficial to the restoration of nerves, and has an important role in innerved bone regeneration. In conclusion, these tactics have been used in OS therapy and have produced the targeted outcomes, laying the groundwork for further advancements in treatment approaches. While these advancements are encouraging and show great potential, there are still some challenges. Although novel preparation methods for BP nanoparticles have been proposed, the high preparation cost and low yield pose challenges to achieving large-scale, high-concentration preparation, which hinders clinical applications. In terms of constructing BP-based DDS, electrostatic interactions are still the most commonly used method for constructing DDS, which are convenient but susceptible to environmental influences, and thus requires clever design, e.g., the construction of environmentally responsive or reversible bonds between the carrier and the drug. In terms of antimicrobial properties, it remains uncertain whether BP-based nanomaterials as antimicrobial drugs will develop drug resistance. Currently, the experimental aspect is only for the study of bacterial culture, while there are not many studies exploring the process of bacterial evolution in depth. So, it is necessary to study the exact mechanism of drug resistance from the level of bacterial proteins and nucleic acids to prevent and control bacterial drug resistance.

Good biocompatibility is an essential characteristic of biomaterials. Various experiments, both *in vitro* and *in vivo*, have been conducted to confirm the biocompatibility of BP. The reviewed experiments consistently demonstrate that BP exhibits good biocompatibility. However, there are conflicting reports possibly due to non-standardized testing protocols or different biological models used. The current challenge lies in the lack of techniques for monitoring the intracellular biodegradation process of BP-based nanomaterials. Therefore, it is necessary to conduct more *in vitro* studies to investigate the relationship between BPNM properties and cell biological outcomes. Besides, further exploration is required to understand how various human disease-associated environments (such as diabetic hyperglycemic microenvironment, tumor microenvironment, osteoporotic microenvironment, immune dysregulation microenvironment) can affect the behavior and toxicological effects of BP-based nanomaterials.

The study of BP nanoparticles for OS therapy is still in its early stages and many questions need to be addressed and improved. ​First, BP nanoparticles are unstable under natural conditions, so further research on BP modification strategies is needed for OS therapy. Second, the insufficient depth of NIR light penetration may limit the clinical use of BP nanoparticles in deeper bone defects, so the development of PH and ultrasound-responsive therapies is a good response strategy. In addition, when combining multiple therapeutic strategies for OS therapy, the order in which the individual strategies occur is the most important consideration, and it is preferable for all therapeutic strategies to work together at the same time in order to minimize the side effects caused by high doses and prolonged irradiation. Furthermore, it has been demonstrated that macrophage absorption of BP nanoparticles stimulates the release of inflammatory cytokines like TNF-α. BP nanoparticles in contact with hemoglobin form a protein crown, inducing an immune response that leads to disruption of the local microenvironment. High concentrations (200 μg mL^-1^) of BPQDs can induce cellular lipid peroxidation and affect cell viability. Therefore, the effectiveness of BP nanoparticles on non-tumor cells during OS therapy needs to be further investigated. Last but not least, all of the studies in this review are laboratory tests and still have a long way to go before they can be introduced into clinical trials in practice. Therefore, addressing the biosafety issues arising from the introduction of nanomaterials into the human body would be of extraordinary clinical value. With the work of the current researchers, it is anticipated that the use of BP nanoparticles in OS therapy would advance. This will provide a scientific basis for conducting clinical trials of BP nanomaterials for OS therapies.
